# Zebrafish *prdm12b* acts independently of *nkx6.1* repression to promote *eng1b* expression in the neural tube p1 domain

**DOI:** 10.1186/s13064-019-0129-x

**Published:** 2019-02-27

**Authors:** Ozge Yildiz, Gerald B. Downes, Charles G. Sagerström

**Affiliations:** 1 Department of Biochemistry and Molecular Pharmacology, University of Massachusetts Medical School, Worcester, 364 Plantation St/LRB815, Worcester, MA 01605 USA; 2 Department of Biology, University of Massachusetts, Amherst, MA 01003 USA

**Keywords:** CRISPR, Dorsoventral patterning, Hindbrain, Spinal cord, Interneuron, Locomotion, Transcription

## Abstract

**Background:**

Functioning of the adult nervous system depends on the establishment of neural circuits during embryogenesis. In vertebrates, neurons that make up motor circuits form in distinct domains along the dorsoventral axis of the neural tube. Each domain is characterized by a unique combination of transcription factors (TFs) that promote a specific fate, while repressing fates of adjacent domains. The *prdm12* TF is required for the expression of *eng1b* and the generation of V1 interneurons in the p1 domain, but the details of its function remain unclear.

**Methods:**

We used CRISPR/Cas9 to generate the first germline mutants for *prdm12* and employed this resource, together with classical luciferase reporter assays and co-immunoprecipitation experiments, to study *prdm12b* function in zebrafish. We also generated germline mutants for *bhlhe22* and *nkx6.1* to examine how these TFs act with *prdm12b* to control p1 formation.

**Results:**

We find that *prdm12b* mutants lack *eng1b* expression in the p1 domain and also possess an abnormal touch-evoked escape response. Using luciferase reporter assays, we demonstrate that Prdm12b acts as a transcriptional repressor. We also show that the Bhlhe22 TF binds via the Prdm12b zinc finger domain to form a complex. However, *bhlhe22* mutants display normal *eng1b* expression in the p1 domain. While *prdm12* has been proposed to promote p1 fates by repressing expression of the *nkx6.1* TF, we do not observe an expansion of the *nkx6.1* domain upon loss of *prdm12b* function, nor is *eng1b* expression restored upon simultaneous loss of *prdm12b* and *nkx6.1.*

**Conclusions:**

We conclude that *prdm12b* germline mutations produce a phenotype that is indistinguishable from that of morpholino-mediated loss of *prdm12* function. In terms of *prdm12b* function, our results indicate that Prdm12b acts as transcriptional repressor and interacts with both EHMT2/G9a and Bhlhe22. However, *bhlhe22* function is not required for *eng1b* expression in vivo, perhaps indicating that other *bhlh* genes can compensate during embryogenesis. Lastly, we do not find evidence for *nkx6.1* and *prdm12b* acting as a repressive pair in formation of the p1 domain – suggesting that *prdm12b* is not solely required to repress non-p1 fates, but is specifically needed to promote p1 fates.

**Electronic supplementary material:**

The online version of this article (10.1186/s13064-019-0129-x) contains supplementary material, which is available to authorized users.

## Background

Appropriate function of the adult nervous system requires the establishment of neural circuits during embryonic development. For such circuits to form properly, neurogenesis has to occur at the right time and place, neurons must migrate to the correct site and they must make appropriate connections. Disruptions to any step in this process result in improper neural circuit formation and such disruptions are thought to underlie many neurodevelopmental disorders – including schizophrenia and autism [1].

The embryonic vertebrate neural tube represents a well-studied system of neural circuit formation where various progenitor types form in distinct domains arrayed along the dorsoventral (DV) axis. These progenitor domains form in response to morphogen gradients – particularly dorsally derived Bone morphogenic protein (BMP) and ventrally derived Sonic hedgehog (Shh; reviewed in [2, 3]). In response to these morphogens, each progenitor domain acquires a unique gene expression profile that initially consists primarily of transcription factors (TFs). Strikingly, TFs unique to one progenitor domain frequently cross-repress the expression of TFs associated with adjacent domains, thereby establishing distinct boundaries that delineate individual progenitor domains along the DV axis. The graded morphogen signal, and the resulting distinct transcriptional programs, leads to the development of sensory neurons in the dorsal domains (pd1-pd5) and interneurons and motor neurons in the ventral domains (pd6-p0, p1, p2, pMN, p3) of the neural tube. Neurons from each of these domains then make connections to establish motor circuits that control the activity of limb and trunk musculature [4].

Many TFs that control establishment of progenitor domains along the DV axis belong to the homeodomain (HD) and basic Helix-Loop-Helix (bHLH) families. For instance, work in mouse and chick indicate that Shh activates genes such as *Nkx6*.*1*, *Nkx6*.2, *Nkx2*.*2*, and *Olig2*, while it represses *Pax3*, *Pax6*, *Pax7*, *Dbx1*, *Dbx2* and *Irx3* [5–13]. These TFs then repress each other’s expression to establish distinct progenitor domains. For instance, *Irx3* and *Olig2* are mutually repressive at the p2/pMN boundary [8, 14] such that loss of *Olig2* leads to a ventral expansion of *Irx3* expression, causing the pMN domain to give rise to V2 interneurons and astrocytes in place of motor neurons and oligodendrocytes [14]. More recently, members of the Prdm TF family have also been implicated in the formation of progenitor domains and the establishment of functional motor circuits (reviewed in [15]). The Prdm family consists of many members (Prdm1–16) that harbor an N-terminal PR domain, as well as a variable number of zinc fingers [16, 17], and that appear to preferentially act in complexes with bHLH TFs [15]. Hence, Prdm13 acts together with Ascl1 to promote formation of GABAergic neurons [18, 19], while Prdm8 interacts with the Bhlhe22 (a.k.a. Bhlhb5) TF to regulate axon outgrowth [20]. Of particular interest, Prdm12 is expressed in the developing CNS of mouse, frog, chick and zebrafish [21–23] – specifically in the p1 domain, which gives rise to V1 interneurons. *Prdm12* deficiency in zebrafish and frog results in loss of *eng1* expression from the p1 domain and animals lacking *prdm12* function demonstrate a defective touch-evoked escape response [22, 23], suggesting that the V1 interneurons are absent. However, key aspects of Prdm12 function remain unclear. First, Prdm12 activity has only been assessed via overexpression and transient knock-down approaches – particularly antisense morpholino oligonucleotides (MOs) – that have recently come under scrutiny as prone to non-specific off-target effects. Furthermore, Prdm12 is suggested to act as a transcriptional repressor, but this is based on overexpression in fish and frog embryos [23, 24] and has not been stringently tested. Here, we generate and characterize the first germline *prdm12* mutants using CRISPR/Cas9 to inactivate zebrafish *prdm12b*. *prdm12b* mutants display embryonic lethality and, in accordance with previous *prdm12b* MO analyses, we find that *prdm12b* mutants exhibit loss of *eng1b* expression in the p1 domain together with an abnormal touch-evoked escape response. We also employ luciferase reporter assays to reveal that Prdm12b acts as a bona fide repressor. This repression requires a conserved zinc finger domain that interacts with the Bhlhe22 TF, but, when we generate a *bhlhe22* germline zebrafish mutant, it displays a normal p1 territory – indicating that *bhlhe22* does not need to act with *prdm12b* for p1 progenitor formation in vivo. Lastly, while *Nkx6.1* is known to repress p1 fates in other systems, we find that *prdm12b* and *nkx6.1* does not form a reciprocally repressive TF pair in the zebrafish. Therefore, instead of the p1 domain taking on a p2 fate, a residual domain with unknown properties persists at the p1 position in *prdm12b* zebrafish mutants.

## Methods

### Zebrafish care

Wild type and mutant zebrafish were raised in the University of Massachusetts Medical School Aquatics Facility. All embryos were staged according to previously described morphological standards [25].

### Generation of CRISPR/cas9 mutant zebrafish lines

We designed single guide RNAs (sgRNA) for the zebrafish *prdm12b*, *bhlhe22* and *nkx6.1* genes (Table 1) using the CHOPCHOP web tool [26]. Each sgRNA was assembled by annealing two single stranded oligonucleotides containing the T7 promoter and the target sequence (Additional file 1) followed by PCR amplification, purification and in vitro transcription using T7 RNA polymerase (Promega) as described previously [27]. A linearized plasmid encoding *cas9* was used for in vitro transcription using the SP6 mMessage mMachine Kit (Ambion) according to the manufacturer’s instructions [28]. *cas9* mRNA and sgRNA was co-injected into 1-cell stage zebrafish embryos at the following concentrations: 150 ng/μL sgRNA plus 200 ng/μL *cas9* mRNA for *prdm12b*, 100 ng/μL sgRNA plus 200 ng/μL *cas9* mRNA for *bhlhe22* and 150 ng/μL sgRNA plus 200 ng/μL *cas9* mRNA for *nkx6.1*. The next day, injected embryos were assayed for sgRNA activity by DNA extraction, PCR amplification, restriction digestion and DNA sequencing (Table 1). Detection of F0 founders was done by crossing sgRNA/*cas9*-injected animals with wildtype zebrafish and screening their offspring for mutagenic events using the diagnostic restriction enzymes listed in Table 1. Confirmed founders were crossed to wildtype animals to raise F1 carriers for each mutant.

**Table 1:** Characteristics of CRISPRs targeting *prdm12b*, *bhlhe22* and *nkx6.1*

Target gene	Start Coordinate	Target sequence	Enzyme	Strand	Mutagenesis Rate^a^	Transmission Rate^b^
*prdm12b*	Chr5:66656496	GCTGGGGGAACACCTGTTCG	Taq1α	+	1/4	71/92 um318 43/79 um319
*bhlhe22*	Chr24:25069884	TTCACACACAAAGATCCGGT	BstYI	–	6/14	24/37 um320
*nkx6.1*	Chr21:17886500	AGTGGAGGATGCTGGTCCAG	AvaII	–	8/12	18/21 um321

^a^The fraction of screened F0 animals that carried a mutagenic event

^b^The fraction of screened F1 animals that were heterozygous for a mutagenic event

### Antisense morpholino oligonucleotide injections

Antisense morpholino oligonucleotides (MOs) were obtained from Gene Tools LLC. MO injections were performed into the yolk of 1-cell stage embryos using 1-2 ng of solution containing dilutions of 3 mM morpholino stock, distilled water and phenyl red. An MO with the sequence 5′-GCAGGCAACACTGAACCCATGATGA-3′ was used to target the *prdm12b* translation start site. This MO was reported previously [22] and our analyses in this manuscript demonstrate that the effects of MO-mediated *prdm12b* knockdown are indistinguishable from the effects of *prdm12b* germ line mutations.

### In situ RNA hybridization

Embryos were fixed in 4% paraformaldehyde (PFA) and stored in 100% methanol at − 20 °C. In situ RNA hybridization was performed as described [29] followed by a color reaction using NBT/BCIP in 10% polyvinyl alcohol. RNA probes for the genes *eng1b, evx1, vsx2, pax3, nkx6.1, dbx1* and *prdm12b* were synthesized as previously described [27]. Embryos were dissected from the yolk and flat mounted in 80% glycerol for imaging on bridged coverslips or sectioned as described [30]. Images were captured using a Nikon Eclipse E600 microscope equipped with spot RT color camera (model 2.1.1). Images were imported into Adobe Photoshop and adjustments were made to contrast, levels, color matching settings and cropping only. All adjustments were made to the entire image.

### Luciferase reporter assays

0.5 × 10^6^ HEK293T cells were seeded in 6-wells plate and cultured in antibiotic free Dulbecco’s Modified Eagle Medium (DMEM; Gibco) supplemented with 10% fetal bovine serum (Hyloclone) overnight. Transient transfections were performed using Lipofectamine 2000 reagent (Invitrogen) according to the manufacturer’s instructions. For each transfection, 200 ng of the pGL4.31[luc2P/GAL4UAS/Hydro] reporter plasmid and 50 ng pRL-SV40 control plasmid was combined with varying concentrations of GAL4DBD expression plasmids (the fusion proteins were cloned into the pCS2 expression plasmid; exact concentrations are given in figure legends). Empty vector DNA was included to keep the total amount of DNA constant for all transfections. Luciferase activity was measured 24 h post transfection and firefly luciferase levels were normalized to *renilla* luciferase levels using the Dual Luciferase Reporter Assay System (Promega) following the manufacturer’s instructions in a Perkin Elmer Envision 2104 Multiplate reader. For Trichostatin A (TSA) treatment, transfected cells were exposed to either DMSO, 50 nM or 250 nM TSA for 12 h starting 24 h after transfection and then harvested for luciferase assays.

### Co-immunoprecipitation and Western blotting

3 × 10^6^ HEK293T were seeded in 10 cm dishes and transfected as above. Transfected cells were lysed in 4 mL of ice-cold co-IP buffer (50 mM Tris-HCl pH 7.5, 150 mM NaCl, 0.2 mM EDTA, 1 mM DTT, 0.5% Triton X100, 1X Complete Protease Inhibitor (Roche)) followed by incubation on ice for 30 min. Cell lysates were centrifuged at 2000 g for 10 min at 4 °C to eliminate cell debris. For immunoprecipitation, 8 μg of the mouse anti-Flag antibody (Sigma-Aldrich, F3165) was used in each sample and incubated at 4 °C overnight. 40 μL of Dynabeads was added in each sample and incubation was done for 4 h at 4 °C. Four washes of 1 mL co-IP buffer was used to eliminate non-specific binding. Lastly, immune complexes were eluted in 80 μL of 1X Laëmmli buffer (Biorad) containing 2.5% beta-mercaptoethanol. Samples were agitated at 95 °C for five minutes prior to Western blotting. Western Blotting was performed using rabbit HA antibody (Abcam, ab9110) as described previously [31].

### Immunocytochemistry

Primary antibodies: mouse 3A10 (1:100; Developmental Studies Hybridoma Bank (DSHB) [32]), mouse F310 (1:100; DSHB [33]), mouse anti-Isl (39.4D5, 1:100; DSHB [34]), mouse 81.5C10 (Hb9; 1:400; DSHB [35]). Alexa Fluro secondary antibodies: 488, 568 goat anti-mouse (both at 1:200; Molecular probes). Embryos were fixed in 4% AB fix (4% paraformaldehyde, 8% sucrose, 1x PBS) overnight at 4 °C. Whole-mount fluorescent labeling was performed as described [36]. Images were captured on either Nikon Eclipse E600 (3A10, Isl1 and Hb9 staining) or a Zeiss LSM700 confocal microscope (F310 staining). Images were imported into Adobe Photoshop and adjustments were made to contrast, levels, color matching settings and cropping only. All adjustments were made to the entire image.

### Behavioral analysis

Escape responses were elicited by a light tap to the head or tail of an embryo with a 3.22/0.16 g of force Von Frey filament. A high-speed digital camera (Fastec Imaging, San Diego, CA) mounted to a 35 mm lens (Nikon, Melville, NY), recorded each response at 1000 frames/s. Computer software generated in the Downes laboratory [37] quantified the head-tail angle for each frame, which was then plotted in Prism. The calculated escape response began in the frame preceding the first movement until movement was no longer observed.

### Genotyping

CRISPR-generated mutant alleles of *prdm12b*, *bhlhe22* and *nkx6.1* were genotyped by Taq1α, BstYI or AvaII restriction digest, respectively, of PCR products amplified from genomic DNA using primers listed in Additional file 2. *prdm12b*^*sa9887*^ mutants were genotyped by sequencing of PCR products amplified from genomic DNA using primers listed in Additional file 2.

Total RNA from 24hpf WT and *bhlhe22* zebrafish whole embryos was extracted with the RNeasy kit (Qiagen) following manufacturer’s instructions. Total RNA was then used in cDNA kit (ThermoFisher Scientific). Wildtype and *bhlhe22* mutant transcripts were identified by sequencing of PCR products amplified from cDNA using primers listed in Additional file 2.

## Results

### Germline disruption of prdm12b blocks eng1b expression in the p1 domain

**Fig. 1:**
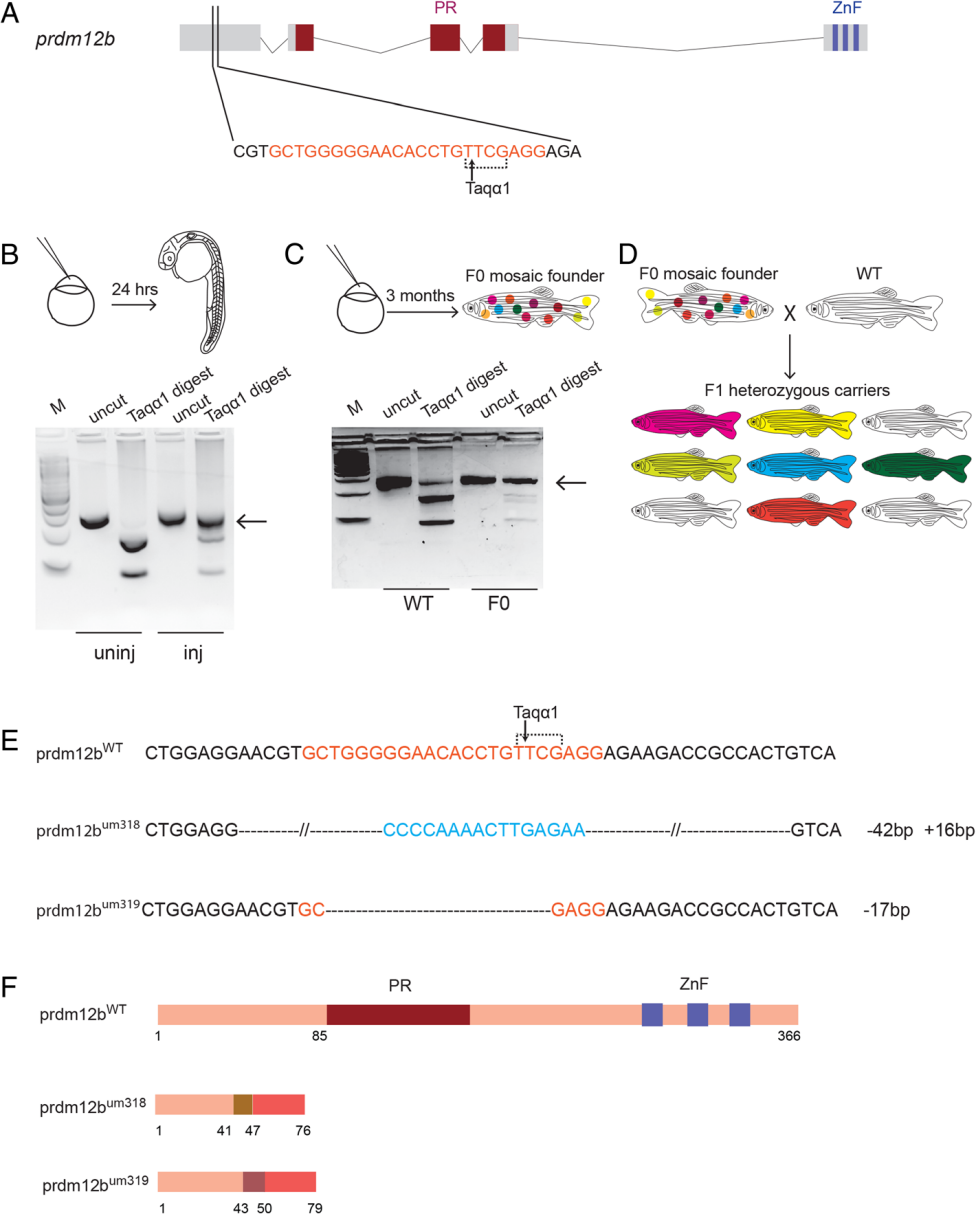
Generation of germ line *prdm12b* mutants. **a**. Schematic showing genomic sequence of *prdm12b*. Exons are indicated as boxes and black lines represent introns. The PR domain and three zinc fingers (ZnF) are highlighted in dark red and blue, respectively. The CRISPR target sequence is shown in red with the Taq α1 restriction site bracketed and the black arrow indicating the Taq α1 cut site. **b**. Identification of functional guide RNAs. sgRNA and *cas9* mRNA was injected into 1-cell stage embryos. Injected embryos were raised to 24hpf and Taq α1 digestion of PCR amplicons from pools of embryos was used to identify CRISPR-induced mutations (black arrow). **c**. Identification of individual F0 founders. sgRNA/*cas9* injected embryos were raised to adulthood and crossed to wildtype fish. Taq α1 digests of PCR amplicons from pools of embryos was used to identify F0 mosaic founders (black arrow). **d**. Identification of F1 animals. Adult F0 mosaic founders were out-crossed to wildtype fish and the F1 offspring raised to adulthood. Taq α1 digests of PCR amplicons from individual fin clip genomic DNA was used to identify heterozygous F1 animals. **e**. Sequencing of F1 genomic DNA revealed the transmission of two different mutant alleles (um318, um319). um318 carries a 42 base pair deletion (black dashes) and a 16 base pair insertion (blue), while um319 carries a 17 base pair deletion (black dashes). The CRISPR target sequence is shown in red. **f**. Predicted amino acid sequence of mutant alleles. The um318 peptide shares its first 41 amino acids, and the um319 peptide its first 43 amino acids, with wildtype Prdm12b. The two mutant peptides then utilize a different reading frame that terminates at a premature stop codon N-terminal to the conserved PR domain. Inj = sgRNA/Cas9-injected embryos, uninj = uninjected control embryos

The *prdm12* TF is known to be expressed in the developing CNS of mouse, chick, *Xenopus* and zebrafish [21–23] – particularly in sensory ganglia and in the p1 domain of the neural tube. The p1 domain gives rise to *eng1b-*expressing V1 interneurons that regulate motor circuits in several vertebrate species [38–40]. Disruption of *prdm12* function using antisense morpholino oligonucleotides (MOs) leads to the loss of *eng1b* expression in the p1 domain, but not in other *eng1b* expressing tissues – such as the midbrain-hindbrain boundary (MHB) and the somites – in zebrafish and *Xenopus* [15, 23], but there have been no germline mutations for *prdm12* produced in any organism. Importantly, recent work has demonstrated several cases where apparently specific MO-derived phenotypes do not match the phenotypes of germ line mutants for the same gene [41]. The underlying causes of such discrepancies are varied, but include off-target effects, as well as compensatory changes in the expression of genes with similar functions to the targeted gene [42]. Hence, it is essential to confirm MO-derived phenotypes by comparisons to the phenotypes of germline mutant animals. To this end, we used the CRISPR/Cas9 genome editing system [43, 44] to generate *prdm12b* germline mutant zebrafish. We tested five sgRNAs targeting the first exon of the *prdm12b* gene and identified one that efficiently disrupts a diagnostic Taqα1 site at position 129 of *prdm12b* exon 1 in 24hpf zebrafish embryos (Fig. 1A, B). Injected embryos were raised to adulthood and screened to identify founders that carry mutations in the *prdm12b* gene (Fig. 1c). In this manner, we identified one mutant F0 founder out of four tested (Table 1). Since zebrafish F0 founders are usually mosaic, this founder was crossed to wildtype fish and the resulting F1 generation raised to adulthood (Fig. 1d). Genotyping revealed that the F0 founder transmitted mutations to 77% (114/171) of its F1 offspring (Table 1). Subsequent sequencing of genomic DNA from individual F1 fish identified two different alleles (*prdm12b*^*um318*^ and *prdm12b*^*um319*^; Fig. 1e, f; Additional file 3). In both alleles, the mutant sequence leads to a frameshift and premature termination of translation upstream of the conserved PR domain and the zinc finger domains. In addition, while we were in the process of generating *prdm12b* mutants, a mutant *prdm12b* allele became available from the zebrafish information resource center (ZIRC) as a product of the zebrafish mutation project (ZMP). This mutant allele (*prdm12b*^*sa9887*^) is ENU-derived and carries a T > C change in an essential splice site at the beginning of intron 2, within the PR domain and upstream of the zinc finger domains (Additional file 4A). We obtained this line from ZIRC and confirmed the presence of the expected mutation by sequencing (Additional file 4B, C).

**Fig. 2:**
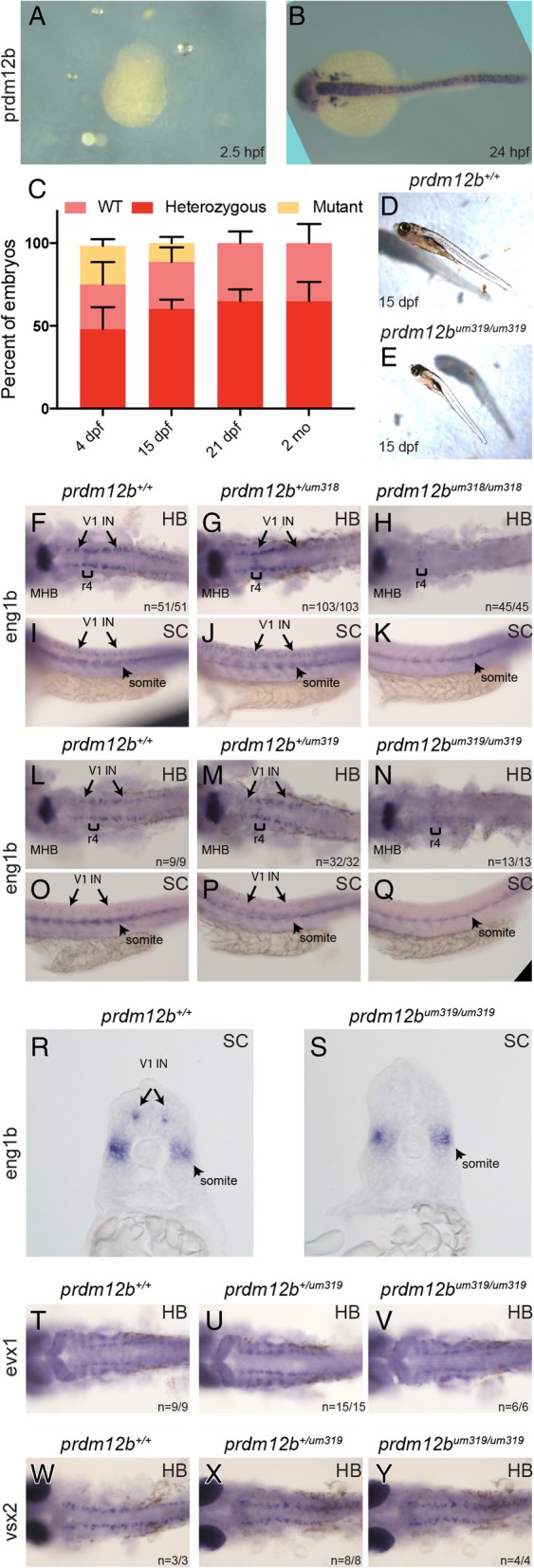
*prdm12b* germ line mutants lack *eng1b* expression in the p1 domain. **a**, **b**. *prdm12b* is not maternally deposited. In situ hybridization detects *prdm12b* expression at 24hpf (**b**), but not at 2.5hpf (**a**), in wildtype embryos. **c**. Bar chart depicting the frequency of each genotype at various time points in broods from crosses of *prdm12b* heterozygous animals. Error bars indicate ±S. E. (*n* = 3). dpf = days post fertilization, mo = months. **d**, **e**. Morphology of 15dpf *prdm12b*^*+/+*^ (**d**) and *prdm12b*^*um319/um319*^ (**e**) fish. **f**-**s**. *eng1b* expression in 24hpf embryos from crosses of *prdm12b*^*+/um318*^ heterozygotes (**f**-**k**), or *prdm12b*^*+/um319*^ heterozygotes (**l**-**s**). Numbers in each panel indicate the fraction of animals with the specified phenotype. **t**, **u**. *evx1* expression in 24hpf embryos from a cross of *prdm12b*^*+/um319*^ heterozygotes. **v**, **w**. *vsx2* expression in 24hpf embryos from a cross of *prdm12b*^*+/um319*^ heterozygotes. Embryos are shown in dorsal (**f**-**h**, **l**-**n**, **t**-**y**) or lateral (**i**-**k**, **o**-**q**) view with anterior to the left, or in cross section (**r**, **s**) with dorsal to the top. Brackets indicate r4, arrows mark V1 interneurons and arrowheads mark somites. MHB = midbrain–hindbrain boundary, HB = hindbrain and SC = spinal cord

Since the effects of MOs wear off as development progresses (largely due to MO degradation) they are not a reliable tool to assess genetic effects on embryo viability. However, having generated *prdm12b* germ line mutants, we were able to examine the effect of *prdm12b* on viability by crossing heterozygous carriers and genotyping the resulting offspring at different stages of embryogenesis. *prdm12b* mRNA does not appear to be maternally deposited (Fig. 2a, b) and is not detected until the end of gastrulation [15], suggesting a relatively late role in development. Accordingly, we observe the expected ~ 25% homozygous *prdm12b* mutants (26/139 for *um318* and 29/116 for *um319*) at 4dpf (Fig. 2c), but by 15dpf only ~ 13% of embryos are homozygous mutant (22/172 for *um319*) and by 21dpf we no longer detect any homozygous mutants (0/129 for *um319*). We also do not observe homozygous mutants when genotyping adult offspring (2 months of age; 0/92 for *um318* and 0/145 for *um319*) from these crosses. Since *prdm12b* mutants start dying between 4dpf and 15dpf, we monitored developing embryos more closely during this time interval and noticed that a fraction of embryos grew at a slower rate (Fig. 2d, e). When the smaller embryos were genotyped, 82% (18/22) turned out to represent homozygous *prdm12b* mutants. This slower rate of growth suggests that the mutants may be unable to feed properly (perhaps due to the motility defects described below). However, when fed brine shrimp, even the mutant embryos show evidence of food in their digestive tract (orange/yellow color in Fig. 2d, e). Hence, the mutants are capable of feeding, although we cannot exclude the possibility that they do so sub-optimally.

Since loss of *eng1b* expression in the p1 domain is the key feature of the zebrafish *prdm12b* morphant phenotype, we next assayed *eng1b* expression in all three *prdm12b* mutant alleles by in situ hybridization at 24hpf. For both CRISP/Cas9-generated alleles, ~ 25% of embryos from crosses of heterozygous carriers lack *eng1b* expression in hindbrain and spinal cord (Fig. 2f-q). In the affected embryos, *eng1b* expression is lost from the p1 domain, but persists at the MHB and in the somites (whole mount in Fig. 2h, k, n, q and section in Fig. 2s). Genotyping revealed that all embryos lacking *eng1b* expression in the p1 domain represent homozygous *prdm12b* mutants (45/45 for *prdm12b*^*um318*^ and 13/13 for *prdm12b*^*um319*^). Similarly, *eng1b* expression is lost in both hindbrain and spinal cord in 27% of embryos from a cross of *prdm12b*^*sa9887/+*^ heterozygous fish, while the remaining embryos show unaffected *eng1b* expression (Additional file 4D-G). We conclude that germ line mutants for *prdm12b* display the same loss of *eng1b* expression as previously reported for *prdm12b* morphants.

### Prdm12b mutant animals display an abnormal escape response

**Fig. 3:**
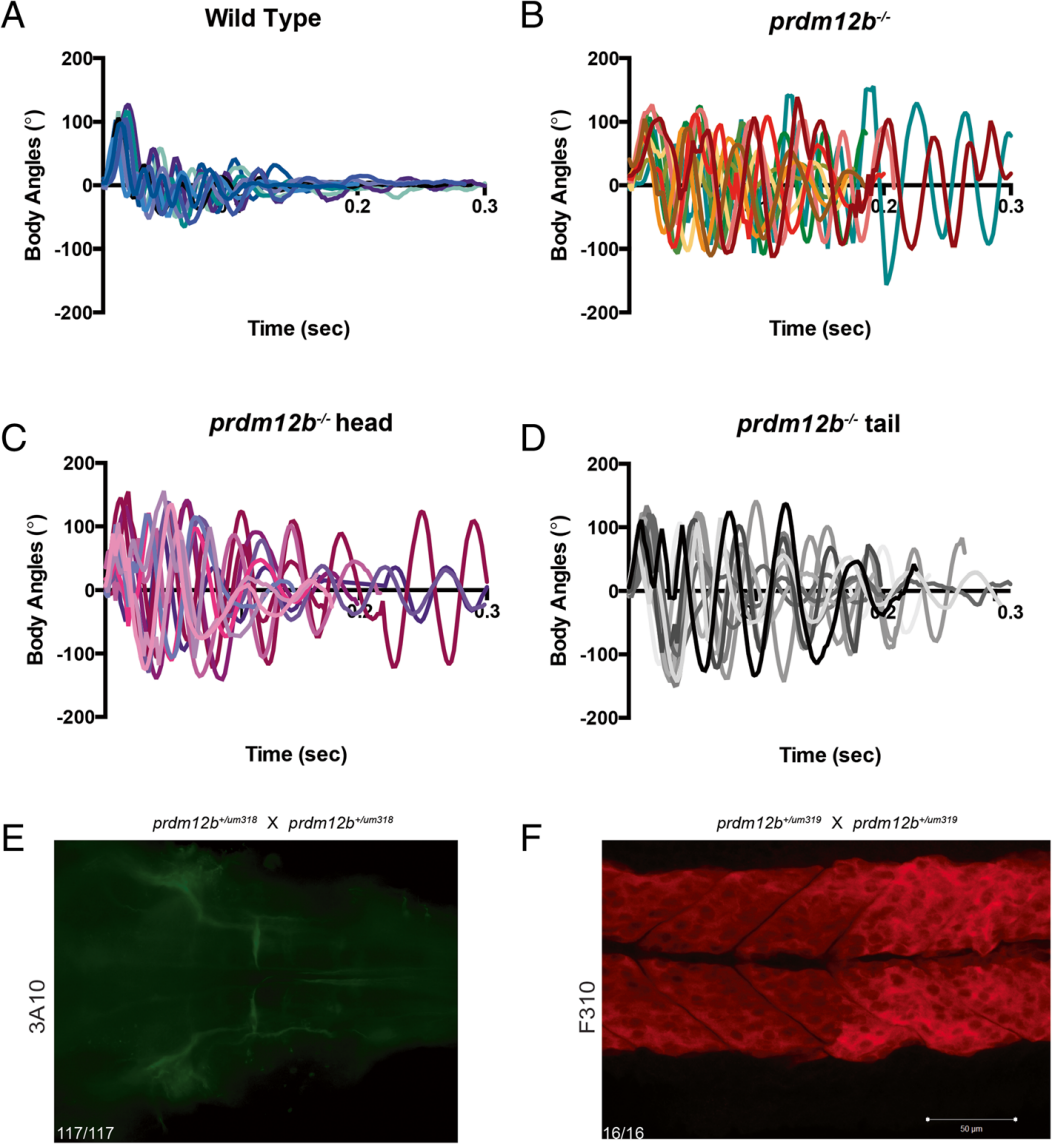
*prdm12b* mutant fish display an abnormal touch evoked response. **a**-**d.** Representative kinematic traces for 10 wildtype (**a**) and 11 *prdm12b* mutant (**b**) fish stimulated with a head touch, as well as for 11 *prdm12b* mutants first assayed with a head touch (**c**) and subsequently with a tail touch (**d**). Zero degrees on the y-axis indicate a straight body while positive and negative angles represent body bends in opposite directions. All fish were at 4dpf. **e**. Anti-3A10 labeling of Mauthner neurons in a cross of *prdm12b*^*+/um318*^ heterozygotes (*n* = 117). **f**. Anti-F310 labeling of somites in a cross of *prdm12b*^*+/um398*^ heterozygotes (*n* = 16)

V1 inhibitory interneurons are responsible for the modulation of motor circuits in many species, including zebrafish, *Xenopus* and mouse ([25, 32], reviewed in [45]). Accordingly, we previously demonstrated that *prdm12b* morphants display abnormal movements in response to touch [15]. The touch-evoked escape response is a classical method of assessing functionality of motor output in aquatic species [46] and it has been applied to zebrafish [47, 48]. In this test, a touch stimulus causes the fish to undergo a large amplitude body bend (C bend), which reorients the animal away from the stimulus. The initial large amplitude body bend is followed by lower amplitude counter bends, allowing the fish to propel itself away. Strikingly, the escape response of *prdm12b* morphants is exaggerated, such that morphants perform not just one, but several repetitive C-bends and, compared to a wild type response – which lasts ~ 100 ms – the response of *prdm12b* morphants is prolonged and may continue for several hundred milliseconds [15]. To determine if this defect is observed also in germline mutants, we assessed the escape response of 4dpf old *prdm12b* mutant fish to a head tap, followed by genotyping. We find that all *prdm12b* mutants (9/9 for *um318* and 8/8 for *um319*), respond by carrying out repetitive C-bends (up to seven C-bends) for extended periods of time (Fig. 3a, b; Additional files 5, 6 and 7). We extended this analysis to also score the response of *prdm12b*^*um319*^ homozygous mutant animals when tapped on the tail. We observed no differences between responses to head versus tail stimulation – in all 11 cases were the responses exaggerated to both stimuli (Fig. 3c, d; Additional files 5, 8, 9). The touch-evoked escape response is mediated via reticulospinal neurons – most notably the Mauthner cells, but also MiD2 and MiD3 cells – and our results therefore indicate that this pathway is abnormal in *prdm12b* mutants. Notably, there is no known circuit connecting V1 interneurons to the reticulospinal cells, suggesting that the abnormal escape response observed in *prdm12b* mutants may be independent of the loss of V1 interneurons. Indeed, the behavior of the mutants is consistent with enhanced or excessive activity of this pathway, perhaps due to impaired synapse function or circuit regulation. Accordingly, we do not detect structural defects in either the morphology of Mauthner cells (Fig. 3e), or the structure of trunk/tail musculature (Fig. 3f). We conclude that *prdm12b* germ line mutant animals display a defective escape behavior that is qualitatively and quantitatively indistinguishable from that of *prdm12b* morphants.

### Prdm12b acts as a repressor in vitro

**Fig. 4:**
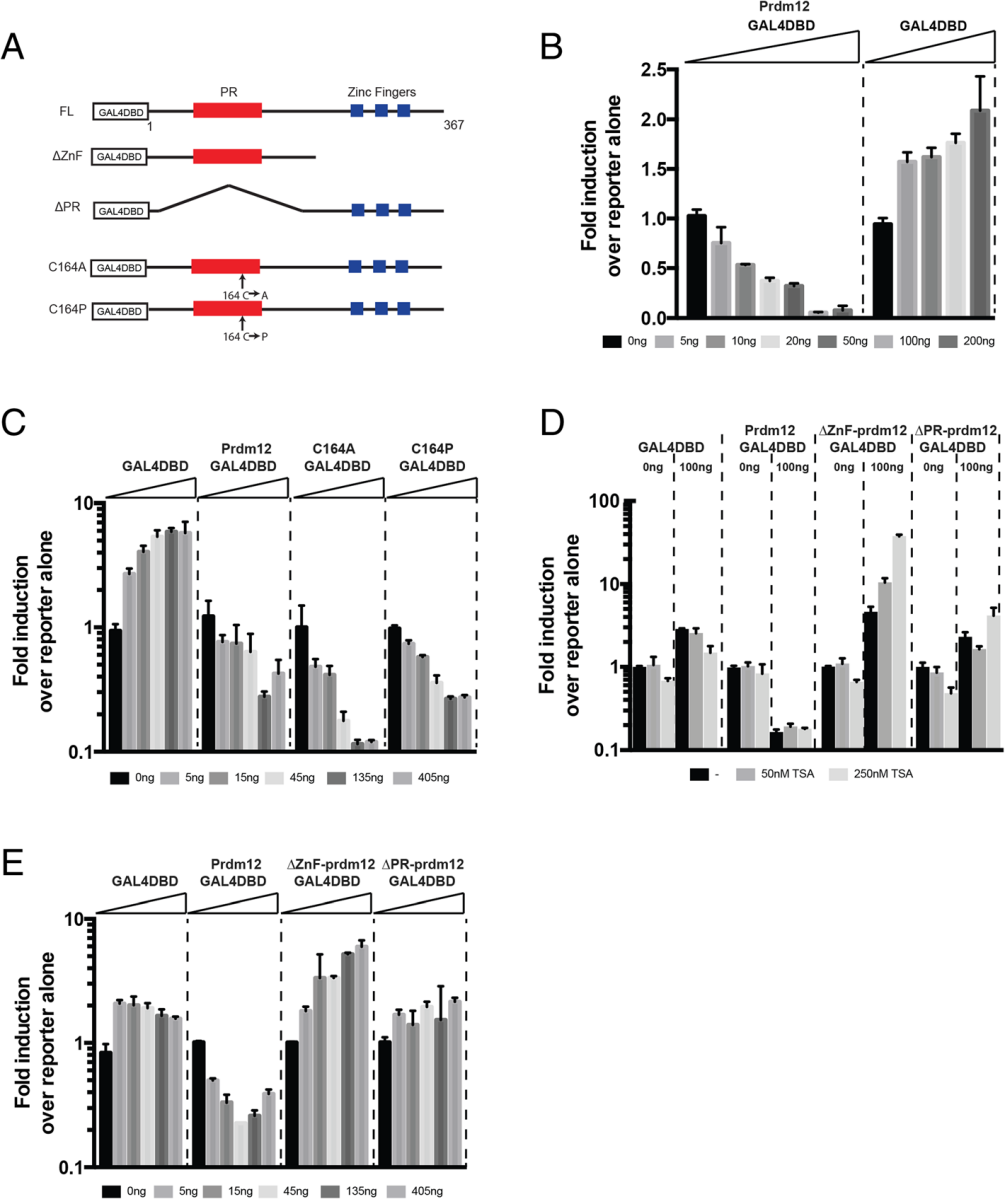
The zinc finger domain is necessary for Prdm12b-mediated repression. **a**. Diagram of GAL4DBD-Prdm12b constructs. FL = full-length, PR = PR domain, ZnF = zinc finger domain. **b**-**e**. Reporter assays in HEK293 cells testing activity of GAL4DBD-Prdm12b constructs. For each experiment, the pRL-SV40 *renilla*-luciferase control plasmid and the pGL4.31 UAS:Firefly-luciferase reporter plasmid were co-transfected with the indicated GAL4DBD-Prdm12b construct or with a plasmid containing the GAL4DBD alone. Each construct was tested in triplicate and luciferase activity is expressed as mean fold induction ± SE over pGL4.31 reporter alone. Transfection efficiency was corrected by normalizing to *renilla* luciferase activity

The fact that *prdm12b* belongs to a family of transcription factors, together with the finding that loss of *prdm12b* function abolishes *eng1b* expression, suggests that this factor may function as a transcriptional activator. Accordingly, transfection of *prdm12* into P19 cells upregulates p27 mRNA and protein levels [49]. However, recent reports instead suggest that *prdm12* acts as a repressor [23], but this conclusion was based on overexpression experiments in vivo and has not been tested directly. To more directly determine whether *prdm12b* acts as an activator or repressor, we made use of classical reporter assays. While *prdm12b* possesses three putative zinc-fingers (ZnFs), it is not clear if these are sufficient for DNA binding and there is no well-defined genomic motif for Prdm12b binding. We therefore fused the well-characterized DNA binding domain (DBD) from the GAL4 transcription factor in-frame to the N-terminus of zebrafish Prdm12b (Fig. 4a; Additional file 10). Transcriptional activity was measured using the pGL4.31 reporter vector that contains multiple GAL4 binding sites (upstream activation sequence; UAS) in front of the firefly luciferase gene. Co-transfection of the reporter plasmid together with the GAL4-DBD alone led to a modest increase in Luciferase activity (Fig. 4b). Strikingly, when the GAL4DBD-Prdm12b fusion protein was instead co-transfected with the reporter plasmid, a dose-dependent reduction in Luciferase activity was observed (Fig. 4b), indicating that the Prdm12b protein functions as a repressor.

Prdm12b contains two types of conserved domains – the PR domain and the zinc fingers. The PR domain is related to SET domains that function as histone lysine methyl transferases (HMTs). Most PR domain proteins lack the H/RxxNHxC motif that is essential for HMT activity [50]; however, Prdm2, Prmd3, Prdm6, Prdm8, Prdm9 and Prdm13 were recently shown to exhibit intrinsic methyltransferase activity [51–55]. Accordingly, the PR domain of Prdm12b has been postulated to act as a H3K9 methyltransferase – to deposit methyl groups onto lysine 9 of histone 3 –thereby repressing gene expression [24]. A recent study of Prdm9 demonstrated that cysteine 321 (Cys^321^) is highly conserved among Prdm family members that have intrinsic histone methyl transferase activity and that substituting Cys^321^ with a proline decreases Prdm9 activity ~ 1000 fold [56]. Our sequence comparison of Prdm1, 9, 10 and 12b revealed that Prdm12b carries a cysteine residue (Cys^164^) at the analogous position to Cys^321^ in Prdm9, while Prdm1 and Prdm10 (that lack methyltransferase activity) contain a proline at this position. To determine the functional contribution of Cys^164^, we tested the activity of several substitution mutants using the luciferase assay, but neither a cysteine - > proline, nor a cysteine - > alanine, substitution at position 164 affected the repressive activity of Prdm12b (Fig. 4c). Deletion of the entire PR domain proved to be uninformative as this protein was unstable in HEK293 cells (Additional file 10). Previous work also demonstrated that some Prdm proteins act as repressors by recruiting histone deacetylases (HDACs) via the PR domain [57–59], but we find that Trichostatin A (TSA; a HDAC inhibitor) does not affect the repressive activity of Prdm12b (Fig. 4d). Lastly, we deleted the conserved zinc fingers in Prdm12b in order to determine if they might be required for its repressive function. Strikingly, deletion of the ZnFs completely abolished the repressive activity of Prdm12b and instead appears to produce a protein with slight activator activity (Fig. 4e). Taken together, our results indicate that Prdm12b functions as a repressor and that this activity requires intact zinc finger domains, at least in the context of a GAL4DBD fusion protein.

### Prdm12b interacts with the Bhlhe22 transcription factor and the EHMT2 methyltransferase

**Fig. 5:**
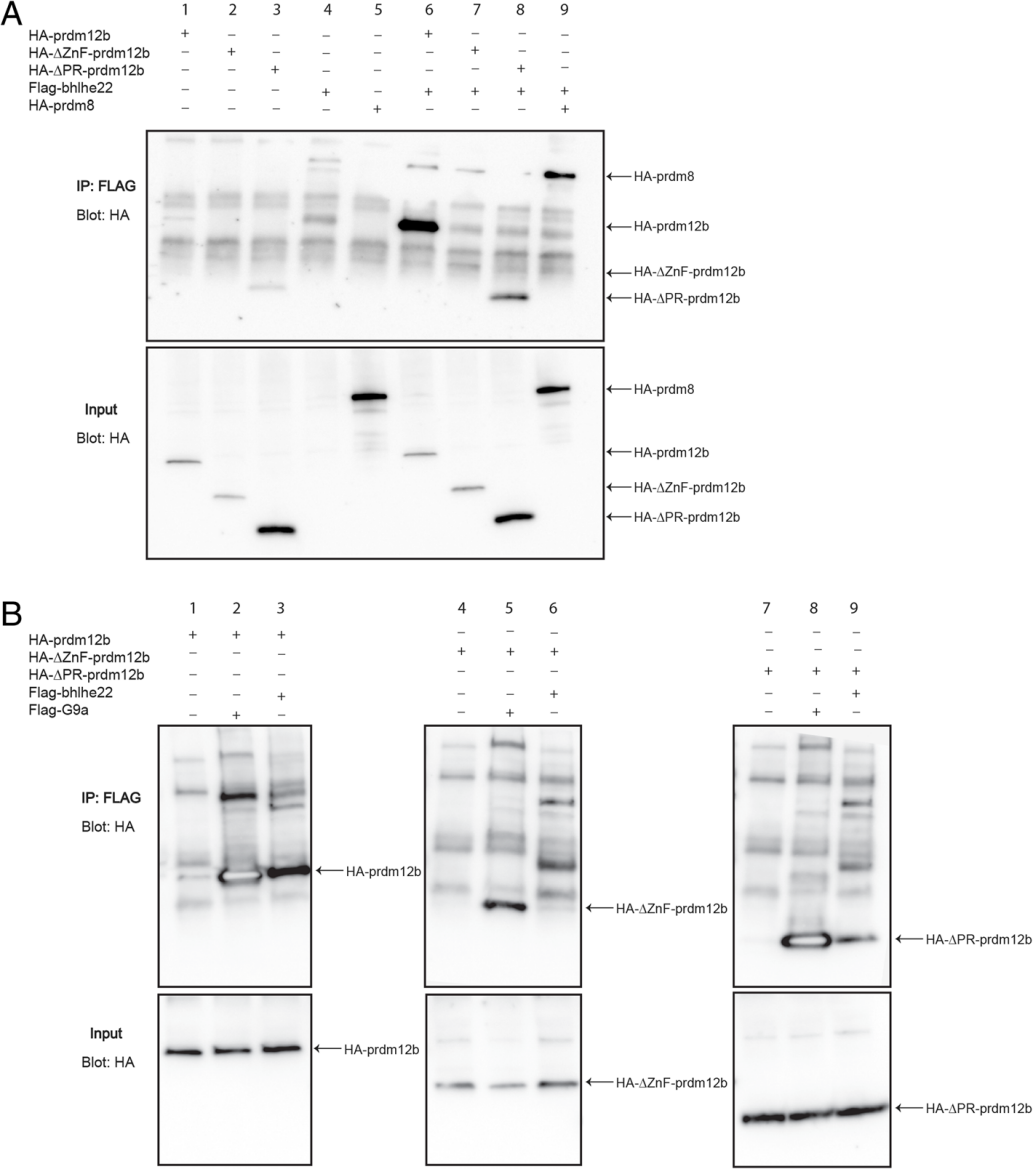
Prdm12b interacts with Bhlhe22 and EHMT2/G9a. **a**, **b**. Co-immunoprecipitation experiments assaying interactions between Prdm12b and Bhlhe22 or EHMT2/G9a. The indicated constructs were co-transfected into HEK293T cells followed by immunoprecipitation with anti-Flag and Western blotting with anti-HA. Arrows at right indicate the expected sizes of each protein. Additional file: 10B demonstrates that Flag-G9a and Flag-Bhlhe22 are stable upon transfection into HEK293 cells

As discussed, it is unclear if Prdm12b binds DNA directly and it may instead be recruited to genomic binding sites by forming complexes with a DNA-binding factor. Since *prdm12b* is expressed only in the p1 domain, we focused our search for DNA-binding Prdm12b-interactors to ones that are co-expressed with *prdm12b* in the p1 domain. Based on this criterion, the Bhlhe22 transcription factor (also known as Bhlhb5) represents a potential binding partner for Prdm12b. In particular, *bhlhe22* is expressed in the pdl6, p1, p2 and p3 domains and has been implicated in the specification of V1 and V2 interneurons [60]. Furthermore, Bhlhe22 has been shown to form complexes with Prdm8, suggesting that it may act broadly as a partner for Prdm proteins [20]. Using co-immunoprecipitation, we confirmed the interaction between Bhlhe22 and Prdm8 (Fig. 5a, lane 9) and further demonstrated robust binding between Bhlhe22 and Prdm12b (Fig. 5a, lane 6). More detailed analyses using Prdm12b deletion constructs indicated that the ZnF domain – that we already identified as necessary for Prdm12b-mediated repression (see Fig. 4d) – is required for Bhlhe22 binding (Fig. 5a, lane 7). In contrast, the PR domain does not appear to be absolutely required for the Prdm12b-Bhlhe22 interaction (Fig. 5a, lane 8).

Moreover, since Prdm12b appears to lack intrinsic methyltransferase activity, it must function by recruiting factors to mediate its repressive effects. Accordingly, Prdm family members recruit various transcriptional repressors ([61–64] and reviewed in [16]). In particular, Prdm1, 5 and 6, as well as Prdm12, have been shown to bind EHMT2/G9a – a H3K9 methyltransferase [57, 59, 65, 66]. In the case of Prdm12, binding to EHMT2/G9a is reportedly mediated by the ZnF domains [49]. Since this is the same domain that we find to be required for binding to Bhlhe22, we examined this in further detail. We confirmed that Prdm12b interacts with EHMT2/G9a (Fig. 5b, lane 2), but find that neither the ZnF, nor the PR domain, is required for this binding (Fig. 5b, lanes 5 and 8).

We conclude that Prdm12b binds to both Bhlhe22 and EHMT2/G9a. Additionally, the Prdm12b ZnF domain – that is essential for Prdm12b-mediated repression – is required for binding to Bhlhe22, but not to EHMT2.

### bhlhe22 is not required for eng1b expression in the zebrafish p1 domain

**Fig. 6:**
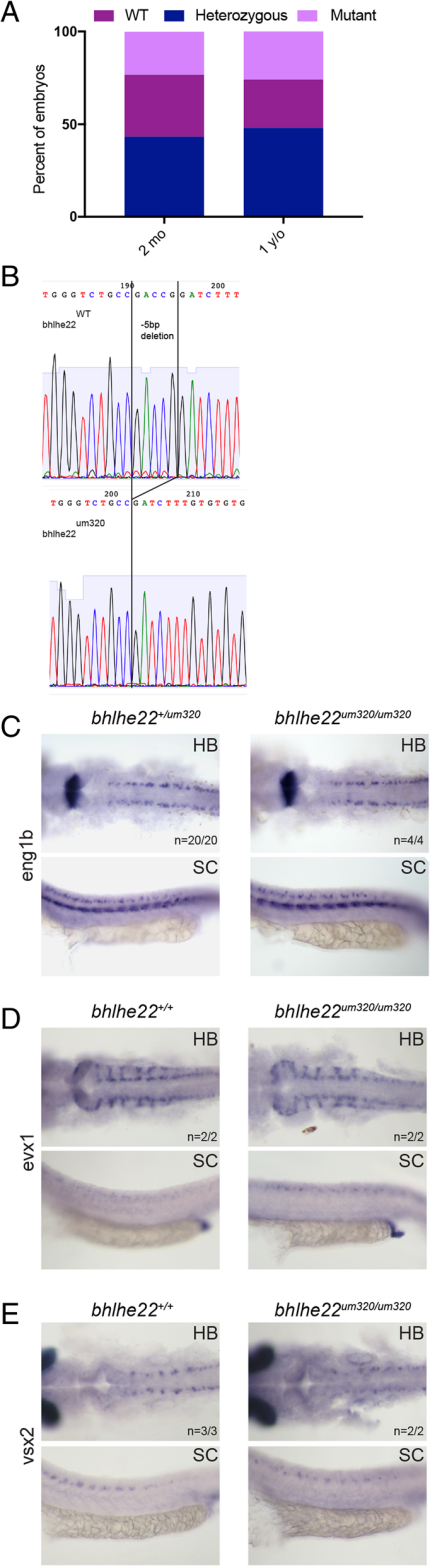
Analysis of *bhlhe22* mutant zebrafish. **a**. Chart depicting the frequency of each genotype at various timepoints in broods from crosses of *bhlhe22*^*+/um320*^ heterozygous fish. mo = month, y/o = year old. **b**. Sequencing traces of transcripts from wild type versus *bhlhe22*^*um320/um320*^ animals showing the expected 5 bp deletion. **c**-**e**. Expression of *eng1b* (**c**), *evx1* (**d**) and *vsx2* (**e**) in 24hpf wildtype and *bhlhe22*^*um320/um320*^ mutant embryos

Previous work reported that siRNA-mediated knock-down of *bhlhe22* in the chick spinal cord leads to a reduction in *eng1* expression in the p1 domain [60], akin to the effect we observe in *prdm12b* mutants. The similarity of the *bhlhe22* and *prdm12b* loss-of-function phenotypes, taken together with our finding that these two proteins form complexes, suggests that *bhlhe22* and *prdm12b* may cooperate to control *eng1b* expression. To test this possibility, we generated germline mutants for zebrafish *bhlhe22* using the CRISPR/cas9 system. Specifically, a sgRNA targeting the 5′ end of the *bhlhe22* coding sequence (that is contained on a single exon) was used to generate six founders carrying mutations in the *bhlhe22* gene (Table 1; Additional file 11A-D). One founder was characterized further and found to transmit a small deletion that introduces a frameshift, which is predicted to cause premature termination of Bhlhe22 protein synthesis upstream of the bHLH domain (Additional file 3B, Additional file 11E). We find that animals homozygous for this mutant allele (*bhlhe22*^*um320*^) are viable to adulthood (Fig. 6a). As expected, sequencing of *bhlhe22* transcripts from such homozygous animals detected only the mutant sequence confirming presence of the mutant allele (Fig. 6b). To test if *bhlhe22* might function with *prdm12b* in p1 formation, we examined *eng1b* expression in *bhlhe22*^*um320*^ animals by in situ hybridization. We find that expression of *eng1b* is unaffected in homozygous *bhlhe22* mutants (Fig. 6c). Since siRNA-mediated knock-down of *bhlhe22* reportedly disrupts gene expression in p0-p2 of chick embryos [60], we also examined expression of *vsx2* in the p2 domain (Fig. 6d) and *evx1* in the p0 domain (Fig. 6e), but do not observe any disruptions. We conclude that, in contrast to the situation in chick, zebrafish *bhlhe22* is not required for p1 domain formation.

### prdm12b does not maintain the p1 domain by repressing nkx6.1

**Fig. 7:**
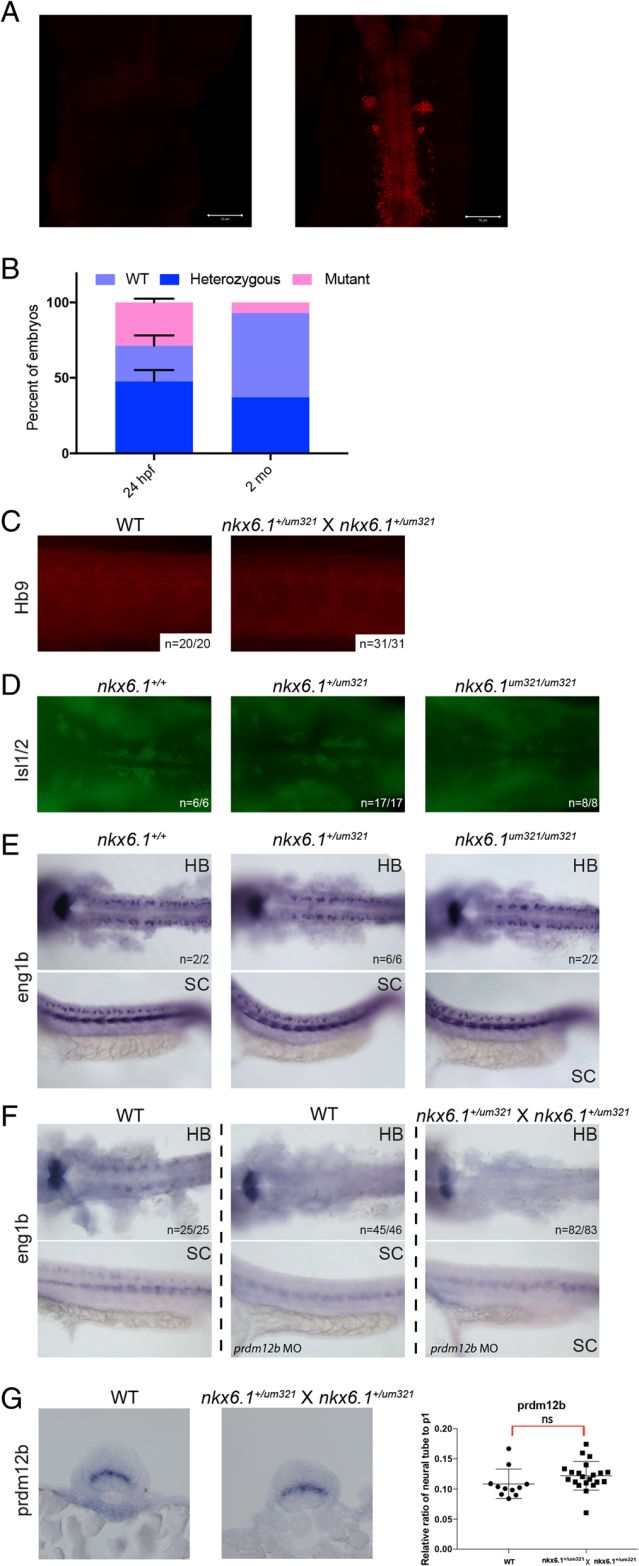
*prdm12b* does not maintain the p1 domain by repressing *nkx6.1*. **a**. Anti-Nkx6.1 immunostaining of *nkx6.1*^*um321/um321*^ mutant (left) and wildtype (right) embryos at 30hpf. **b**. Chart indicating the frequency of each genotype at various time points in broods from crosses of *nkx6.1*^*+/um321*^ heterozygous mutants. **c**. Hb9 immunostaining in wildtype (left) versus a cross of *nkx6.1*^*+/um321*^ heterozygous embryos (right) at 33hpf. **d**. Islet-1/2 immunostaining of 50hpf embryos from a cross of *nkx6.1*^*+/um321*^ heterozygotes. **e**. Expression of *eng1b* in 24hpf embryos from a cross of *nkx6.1*^*+/um321*^ heterozygotes. **f**. Expression of *eng1b* in 24hpf uninjected wildtype embryos (left panels), 24hpf *prdm12b* MO-injected wildtype embryos (middle panels) and 24hpf *prdm12b* MO-injected embryos from a cross of *nkx6.1*^*+/um321*^ heterozygotes (right panels). **g**. Expression of *prdm12b* in a representative wildtype embryo (left panel) and a representative embryo from a cross of *nkx6.1*^*+/um321*^ heterozygotes (middle panel) at 24hpf. Right panel shows quantification of the size of the *prdm12b* expression domain in 11 wildtype embryos and 20 embryos from a cross of *nkx6.1*^*+/um321*^ heterozygotes. Numbers in panels indicate the fraction of embryos displaying the phenotype shown

Repressive interactions are common during formation of the neural tube, whereby mutually repressive pairs of TFs are involved in the establishment of individual progenitor domains (reviewed in [2, 15]). Since *prdm12b* appears to act as a repressor, it is plausible that it forms a repressive pair with *nkx6.1* to establish the p1 domain and permit *eng1b* expression. Accordingly, *nkx6.1* mutant mice display a ventral expansion of the p1 domain at the expense of the p2, pMN and p3 domains [67]. Furthermore, dorsal expansion of *nkx6.1* has been reported in *prdm12* MO-injected fish and frog embryos [22, 23] and overexpression of *prdm12* inhibits *nkx6.1* expression in frog embryos. To test this model further, we generated *nkx6.1* mutant zebrafish by targeting a sgRNA to the 5′ end of exon 1. This produced eight founders carrying mutations in the *nkx6.1* gene (Table 1; Additional file 3C; Additional file 12). Five of these were characterized further and found to transmit two different mutant alleles. The *nkx6.1*^*um321*^ allele contains a 23 bp deletion while the *nkx6.1*^*um322*^ allele carries a 1 bp insertion (as well as three single base pair substitutions). In both alleles, this leads to frameshifts that terminate at a premature stop codon upstream of the HOX domain. Accordingly, immunostaining with an anti-Nkx6.1 antibody revealed loss of Nkx6.1 protein in homozygous *nkx6.1*^*um321/ um321*^ mutants (Fig. 7a). Similar to the situation with *prdm12b* mutants, we find that homozygous *nkx6.1*^*um321*^ mutant animals are observed at the expected ratio during early development, but we detect only a few homozygous *nkx6.1*^*um321*^ animals at adulthood (Fig. 7b). While *nkx6.1* mutant mice display a profound loss of motor neurons [67], *nkx6.1* MO-injected zebrafish show defective formation in only a subset of motor neurons and only at later stages of development [68, 69]. In general agreement with these MO-based zebrafish studies, we do not detect overt changes in expression of the *hb9* motor neuron marker in *nkx6.1* mutant zebrafish (Fig. 7c), but we do observe subtle defects in the formation of branchiomotor neurons in the hindbrain (Fig. 7d).

**Fig. 8:**
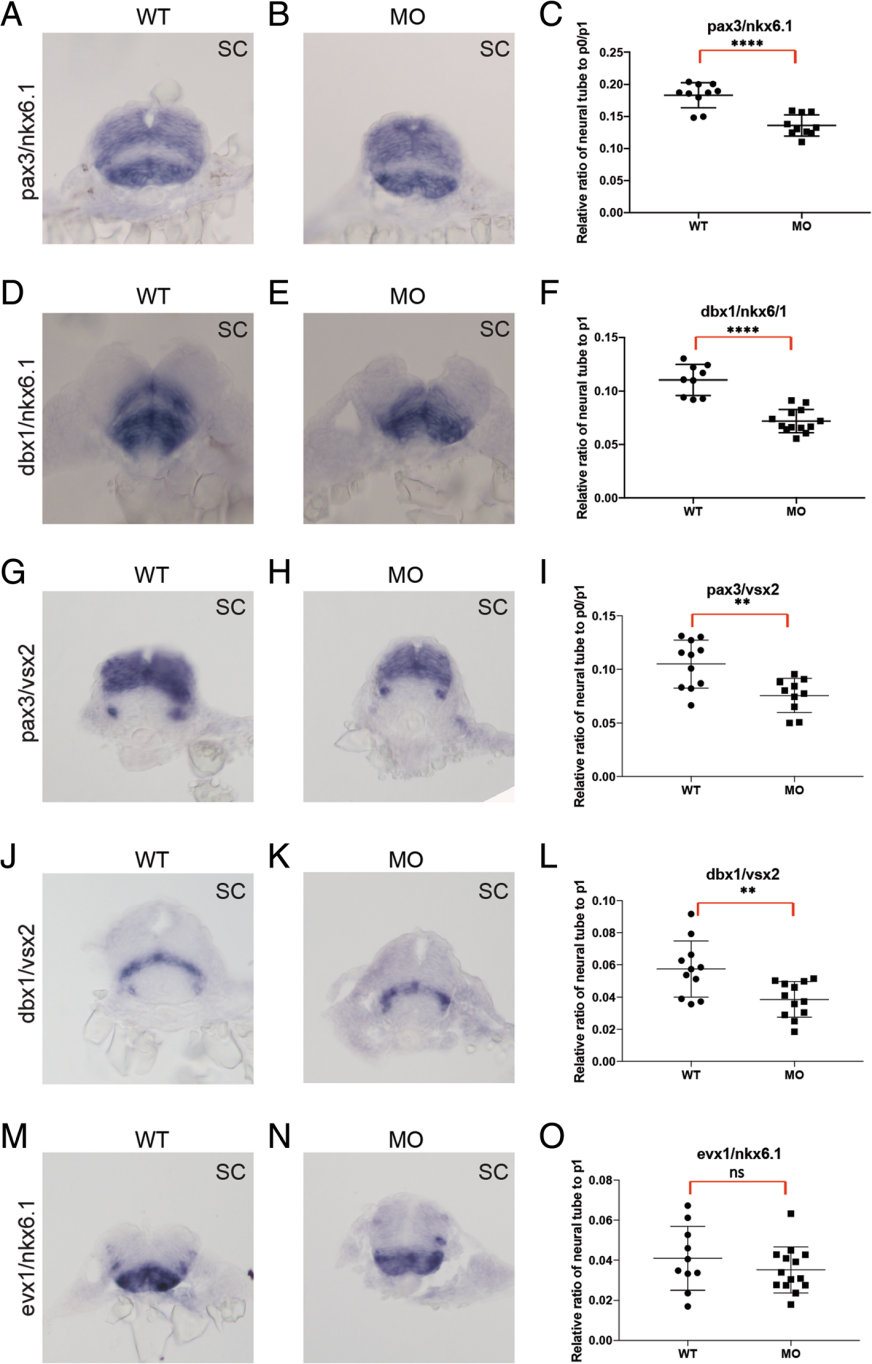
*prdm12b* controls the size of the p1 domain. Expression of *pax3/nkx6.1* (**a**, **b**), *dbx1/nkx6.1* (**d**, **e**), *pax3/vsx2* (**g**, **h**), *dbx1/vsx2* (**j**, **k**) and *evx1/nkx6.1* (**m**, **n**) in 24hpf wildtype (**a**, **d**, **g**, **j**, **m**) or *prdm12b* MO-injected (**b**, **e**, **h**, **k**, **n**) embryos. Panels show cross sections through the spinal cord with dorsal to the top. **c**, **f**, **i**, **l**, **o** show quantification of the size (along the dorsoventral axis) of the p0/p1 domain (**c**, **i**) or the p1 domain (**f**, **l**, **o**) relative to the neural tube. At least 10 representative sections were used for each gene pair

We next used the *nkx6.1* mutant fish to test if *nkx6.1* and *prdm12b* act as a repressive pair to establish the p1 domain and enable *eng1b* expression. However, we do not find evidence for expansion of the *eng1b* (Fig. 7e) or *prdm12b* (Fig. 7g) expression domains in *nkx6.1* mutants. In accordance with previous reports, we observe a slight expansion of the *nkx6.1* domain in *prdm12b* loss of function animals, but this effect falls below the level of statistical significance (Additional file 12G). Furthermore, if *nkx6.1* and *prdm12b* act as a repressive pair, *nkx6.1* would expand into the p1 domain in *prdm12b* mutant animals, thereby expanding the p2 domain at the expense of the p1 domain and leading to loss of *eng1b* expression. Therefore, we would expect *eng1b* transcripts to be present in the p1 domain of animals lacking both *nkx6.1* and *prdm12b* function. To test this, we microinjected the *prdm12b* MO (that we know phenocopies the *prdm12b* germ line mutant; see Figs. 1, 2 and 3 [22]) into embryos from a cross of heterozygous *nkx6.1*^*um321*^ carriers. We find that *eng1b* expression is absent in all MO-injected embryos, regardless of *nkx6.1* status (Fig. 7f), indicating that loss of *eng1b* expression is not the result of *nkx6.1*-mediated expansion of the p2 domain. Lastly, if the loss of *eng1b* expression in *prdm12b* mutants is due to expansion of adjacent domains, we would expect the p1 domain to be absent in *prdm12b* loss of function animals. Using five different combinations of domain-specific genes as markers, we find that the p1 domain is significantly smaller, but still present, in the absence of *prdm12b* function (Fig. 8a-o). We conclude that *prdm12b* is required for establishing an appropriately sized p1 domain, not for preventing *nkx6.1-*mediated dorsal expansion of adjacent domains.

## Discussion

We report the first germline mutants disrupting function of the *prdm12* TF. In particular, we find that three distinct zebrafish *prdm12b* mutant alleles produce an identical phenotype. We use these lines to extend previous characterization of *prdm12* loss of function animals to demonstrate that *prdm12b* is essential for embryonic development, specifically for formation of the neural circuit controlling a classical escape response. Using in vitro approaches, we further demonstrate that Prdm12b functions as a bona fide transcriptional repressor – most likely by recruiting EHMT2/G9a. Although Prdm12b binds via its essential zinc-finger domain to the Bhlhe22 TF, generating and analyzing a *bhlhe22* germline zebrafish mutant revealed no effects on *eng1* expression in the p1 domain – indicating that *prdm12b* and *bhlhe22* do not need to act together for p1 formation in vivo. Lastly, it has been suggested that *prdm12b* and *nkx6.1* form a cross-repressive TF pair essential for the establishment of p1 domain fates. We tested this hypothesis by generating a *nkx6.1* germline zebrafish mutant and analyzing it along with our *prdm12b* mutant, but do not find support for such a cross-repressive arrangement. In fact, instead of the p1 domain taking on a p2 fate in *prdm12b* mutants, a domain persists at the p1 position, but it does not express genes indicative of a specific progenitor class.

### prdm12b germ line mutants recapitulate the phenotype observed using antisense-based approaches

Prdm12 function has been addressed previously, but only by transient loss of function approaches. In particular, antisense morpholino oligos (MOs) were first used in zebrafish [22] and subsequently in frog [23, 24] to disrupt *prdm12* function. The resulting animals lack expression of *eng1* in the p1 domain of the neural tube, but gene expression appears relatively normal in adjacent domains. *eng1*-expressing progenitors in the p1 domain are known to give rise to V1 interneurons that act in motor circuits (reviewed in [45]). Accordingly, fish and frogs lacking *prdm12* function display abnormal escape responses [22, 23], but the nature of this effect (excessive C-bends) suggests a defect in a reticulospinal cell-controlled circuit that is likely independent of the loss of V1 interneurons. Importantly, recent work has highlighted significant concerns with MO-based approaches. In particular, there are many instances where germ line mutations do not confirm previous reported MO-based phenotypes [41]. While some of these cases may be explained by underappreciated compensatory mechanisms [70], there are striking examples of MO phenotypes that turn out to be due to non-specific or off-target effects [27]. Against this background, it is essential to determine the phenotype of *prdm12* germline mutants. To address this, we used CRISPR/Cas9 to generate two lines carrying frameshift mutations in the zebrafish *prdm12b* gene and also obtained an ENU-induced splice-site mutation from the zebrafish resource center. All three lines display a phenotype that is in good agreement with MO-derived data. In particular, germline mutants lack *eng1b* expression and display escape response defects indistinguishable from those in MO injected embryos. Hence, our findings indicate that, in this case, the various MOs act specifically. Since there is currently no available *prdm12* knockout line in mouse, it remains possible that there will be species-specific differences in *prdm12* function, as was recently observed when comparing MO-injected, zebrafish germ line mutants and mouse germ line mutants of the PG1 *hox* genes [71].

### prdm12b is a bona fide transcriptional repressor

The Prdm12 TF has been suggested to act as a repressor based on overexpression studies in vivo and in dissected frog embryos [23, 24], but as an activator based on transfection experiments in P19 cells [49]. To address this discrepancy, we made use of classical reporter assays and find that zebrafish Prdm12b efficiently represses expression from a luciferase reporter gene. Other members of the Prdm family have been reported to act as repressors, but appear to use distinct mechanisms to do so. For instance, several Prdm TFs recruit histone deacetylases (HDACs) to repress transcription, but we find that an HDAC inhibitor does not affect the repressive properties of Prdm12b, indicating that it functions independently of HDACs. Overexpression of Prdm12 also promotes the deposition of repressive methyl marks on H3K9 [23, 24, 49]. Accordingly, the PR domain of some Prdm proteins exhibits methyltransferase activity and this domain is required for Prdm12 function in *Xenopus* [23]. However, we find that mutating a key conserved PR domain residue does not affect the repressive activity of *prdm12b.* Accordingly, in vitro analyses using core histone substrates failed to detect intrinsic methyltransferase activity for Prdm12 [49]. Notably, murine Prdm12 binds EHMT2/G9a (an H3K9 methyltransferase; [49]) and EHMT2/G9a is required for Prdm12 function in *Xenopus* [23], suggesting that Prdm12 may act as a repressor by recruiting EHMT2/G9a. We show that zebrafish Prdm12b also binds EHMT2/G9a, but in contrast to the situation in the mouse, the Prdm12b zinc finger domains are not required for this interaction.

In spite of the presence of several zinc finger domains, many Prdm proteins require interactions with other TFs for targeting to genomic binding sites. In particular, several Prdm proteins form complexes with bHLH TFs [15]. For instance, Bhlhe22 is known to interact with Prdm TFs [20] and is required for expression of *eng1* in the chick neural tube [60], making it a candidate interaction partner for Prdm12b. Indeed, we show by co-immunoprecipitation that Prdm12b and Bhlhe22 can form a complex. Furthermore, this interaction requires the Prdm12b zinc finger domain that we find is required for Prdm12b repressor activity. To test the role for *bhlhe22* in vivo, we used CRISPR/Cas9 to generate a germline mutant in zebrafish, but we do not find any evidence that *bhlhe22* is required for formation of the p1 domain in zebrafish embryos. It is not clear why loss of *bhlhe22* function produces different effects in zebrafish versus chick, but this may stem from the different approaches used – germline mutation in zebrafish versus transient siRNA-mediated knock-down in chick. The lack of a phenotype may also be the effect of compensatory mechanisms, either by other bHLH TFs - which are broadly expressed in the neural tube [72] – or by more general mechanisms operating to suppress the effects of genetic lesions [73]. We conclude that Prdm12b acts as a repressor of transcription – most likely by recruiting EHMT2/G9a – and that the Prdm12-mediated induction of genes such as p27 is most likely the result of indirect events.

### An undefined domain persists at the p1 position in prdm12b mutants

The mechanism whereby *prdm12* promotes formation of the p1 domain remains unclear. Mutual repression between TFs expressed in adjacent domains is the predominant mechanism for the creation of distinct domains along the dorsoventral axis of the vertebrate neural tube. Since *prdm12* functions as a repressor, it is possible that it acts to repress the formation of adjacent domains. Indeed, overexpression and MO-based approaches in the frog have led to the suggestion that *prdm12* and *nkx6.1* (that is expressed in the p2, p3 and pMN domains) forms such a cross-repressive pair [23]. In this model, loss of *prdm12* would lead to loss of *eng1* expression due to *nkx6.1* expression (and p2 fates) expanding into the p1 domain. However, our initial analyses of *nkx6.1* mutant zebrafish do not support this model. First, if *prdm12b* is required for *eng1* expression in the p1 domain due to its repression of *nkx6.1*, *eng1b* should be restored to the p1 domain in embryos lacking both *nkx6.1* and *prdm12b*, but this is not what we observe. Second, if *prdm12b* and *nkx6.1* cross-repress each other’s expression, *prdm12b* expression should expand ventrally in *nkx6.1* mutants and vice versa, but this also does not occur. Lastly, when one member of a cross-repressive pair is mutated, the corresponding progenitor fate is usually replaced by the adjacent fate, but this is not the case in *prdm12b* mutants – where a domain persists at the p1 position, albeit in a narrower form. Since this domain does not express any of the genes diagnostic for various fates along the DV axis, its exact state is not clear. We note that *prdm12* is reported to have anti-proliferative activity [49] and that p1 progenitor cells must exit the cell cycle prior to differentiating into V1 interneurons. It is therefore possible that *prdm12* is required for this transition and that loss of *prdm12* leaves cells in a proliferative progenitor state.

## Conclusion

Our results demonstrate an essential role for *prdm12b* in zebrafish neurogenesis. By generating germline mutations, we show that a loss of function *prdm12b* allele results in lack of *eng1b*-expressing V1 interneurons, defective Mauthner cell-dependent locomotion – which is indistinguishable from *prdm12b* morphants – and ultimately embryonic lethality. Further analyses revealed that the Prdm12b zinc finger domain, which is essential for repression, is also necessary for binding to the Bhlhe22 TF, but not to EHMT2/G9a. We generated a *bhlhe22* mutant zebrafish line, but find no evidence for *bhlhe22* function in the formation of the p1 domain in zebrafish embryos. Lastly, upon examination of cross-repressive interaction between *prdm12b* and *nkx6.1*, we do not find evidence for *nkx6.1* and *prdm12b* acting as a repressive pair in the formation of the p1/p2 boundary. Our results suggest that *prdm12b* does not only regulate *eng1b* expression in the p1 domain, but also takes part in regulating the size of this domain.

## Additional files

Additional file 1: Sequences of oligos used to generate CRISPR guide RNAs. Detailed features of the oligos used as templates for each guide RNA. (DOCX 13 kb)

Additional file 2: Sequences of primers used to genotype mutant lines. Detailed features of primers used to genotype mutant lines. For *bhlhe22*, the *bhlhe22–1* and *bhlhe22–2* primers were used to amplify genomic DNA while the *bhlhe22–3* and *bhlhe22–4* primers were used to amplify cDNA. (DOCX 13 kb)

Additional file 3: Sequence of mutant *prdm12b*, *bhlhe22* and *nkx6.1* alleles. The predicted amino acid sequence for each mutant allele was aligned to the corresponding wildtype sequence using Clustal Omega. (PDF 236 kb)

Additional file 4: Characterization of the *prdm12b*^*sa9887*^ mutant. a. Schematic showing genomic sequence of *prdm12b*. Exons are indicated as boxes and black lines represent introns. The PR domain and three zinc fingers (ZnF) are highlighted in dark red and blue, respectively. The black arrow indicates a single base pair change in the second intron of *prdm12b*^*sa9887*^. b, c. Sequence traces confirming the expected single nucleotide change in wildtype (b) versus *prdm12b*^*+/sa9887*^ (c) animals. d-g. *eng1b* expression in 24hpf embryos from a cross of *prdm12b*^*+/sa9887*^ animals. Embryos are shown in dorsal (d, e) or lateral (f, g) view with anterior to the left. *eng1b* expression is lost in 27% of embryos compared to 73% of embryos showing wildtype *eng1b* staining. (PDF 1962 kb)

Additional file 5: Detailed analysis of the touch-evoked escape response in *prdm12b* mutant and wild type animals. a, b. Representative kinematic traces of individual wild type (a) and *prdm12b* mutant (B) animals stimulated with a head tap (from Fig. 3a, b). c. Quantification of number of body bends with an amplitude similar to the C-bend (defined as exceeding 100°; from data collected in Fig. 3a, b). d. Quantification of C bend duration (from data collected in Fig. 3a, b). e, f. Representative kinematic traces of individual *prdm12b* mutant animals stimulated with a head (left panels) or a tail (right panels) tap (from Fig. 3c, d). (PDF 837 kb)

Additional file 6: Movie of wild type touch-evoked response. Movie of representative wild type animal tapped on the head (from Fig. 3a; recorded at 1000 frames/second). (MP4 794 kb)

Additional file 7: Movie of *prdm12b* mutant touch-evoked escape response. Movie of representative *prdm12b* mutant animal tapped on the head (from Fig. 3b; recorded at 1000 frames/second). (MP4 1842 kb)

Additional file 8: Movie of *prdm12b* mutant touch-evoked escape response. Movie of representative *prdm12b* mutant animal tapped on the head (from Fig. 3c; recorded at 1000 frames/second). (MP4 771 kb)

Additional file 9: Movie of *prdm12b* mutant touch-evoked escape response. The same *prdm12b* mutant animal as in Additional file 8: was instead tapped on its tail (from Fig. 3d; recorded at 1000 frames/second). (MP4 1040 kb)

Additional file 10: Expression of GAL4DBD-Prdm12b constructs used in transfection experiments. a. Immunoblot showing expression of GAL4DBD-Prdm12b constructs in transfected HEK293T cells. All constructs are stable except Myc-GAL4-∆PR-prdm12b. b. Immunoblot showing expression of Myc-Flag-G9a and Myc-Flag-Bhlhe22 constructs in transfected HEK 293 T cells. (PDF 619 kb)

Additional file 11: Generation of *bhlhe22* germline mutant. a. Schematic showing genomic sequence of *bhlhe22* with the bHLH domain indicated in blue. Note that *bhlhe22* is contained on a single exon. The CRISPR target sequence is shown in red with the BstYI restriction site bracketed and the black arrow indicating the BstYI cut site. b. Identification of functional guide RNAs. sgRNA and *cas9* mRNA was injected into 1-cell stage embryos. Injected embryos were raised to 24hpf and BstYI digestion of PCR amplicons from pools of embryos was used to identify CRISPR-induced mutations (black arrow). c. Identification of individual F0 founders. sgRNA/*cas9* injected embryos were raised to adulthood and crossed to wildtype fish. BstYI digests of PCR amplicons from pools of embryos was used to identify F0 mosaic founders (black arrow). d. Identification of F1 animals. Adult F0 mosaic founders were out-crossed to wildtype fish and the F1 offspring raised to adulthood. BstYI digests of PCR amplicons from fin clip genomic DNA was used to identify heterozygous F1 animals. e. Sequencing of F1 genomic DNA revealed the transmission of one mutant allele (um320) carrying a 5 base pair deletion (black dashes). The CRISPR target sequence is shown in red. f. Predicted amino acid sequence of mutant allele. The um320 peptide shares its first 67 amino acids with the wildtype protein before going out of frame and terminating at a premature stop codon N-terminal to the bHLH domain. (PDF 485 kb)

Additional file 12: Generation of germ line *nkx6.1* mutants. a. Schematic showing genomic sequence of *nkx6.1* with the homeodomain indicated in green. The CRISPR target sequence is shown in red with the AvaII restriction site bracketed and the black arrow indicating the AvaII cut site. b. Identification of functional guide RNAs. sgRNA and *cas9* mRNA was injected into 1-cell stage embryos. Injected embryos were raised to 24hpf and AvaII digestion of PCR amplicons from pooled embryos was used to identify CRISPR-induced mutations (black arrow). c. Identification of individual F0 founders. sgRNA/*cas9* injected embryos were raised to adulthood and crossed to wildtype fish. AvaII digests of PCR amplicons from pools of embryos was used to identify F0 mosaic founders (black arrow). d. Identification of F1 animals. Adult F0 mosaic founders were out-crossed to wildtype fish and the F1 offspring raised to adulthood. AvaII digests of PCR amplicons from fin clip genomic DNA was used to identify heterozygous F1 animals. e. Sequencing of F1 genomic DNA revealed the transmission of two mutant alleles (um321, um322). um321 carries a 23 base pair deletion (black dashes) while um322 carries a 1 base pair insertion (green) and 3 base pair substitutions (blue). The CRISPR target sequence is shown in red. f. Predicted amino acid sequence of mutant alleles. The um320 and um321 peptides share their first 44 amino acids with the wildtype sequence before going out of frame and terminating at a premature stop codon N-terminal to the conserved homeodomain. g. Quantification of the size (along the dorsoventral axis) of the *nkx6.1* expression domain in *prdm12b* MO-injected embryos (data from Fig. 8). (PDF 651 kb)

## Abbreviations

bHLH: Basic Helix-Loop-Helix
CNS: Central nervous system
CRISPR: Clustered Regularly Interspaced Short Palindromic Repeats
DBD: DNA binding domain
dpf: Days post fertilization
DV: Dorsoventral
ENU: N-ethyl-N-nitrosourea
HD: Homeodomain
HDAC: Histone deacetylase
HMT: Histone methyl transferase
hpf: Hours post fertilization
MHB: Mid-hindbrain boundary
MO: Morpholino
sgRNA: Single guide RNA
Shh: Sonic hedgehog
TF: Transcription factor
TSA: Trichostatin A
WT: Wildtype
ZIRC: The Zebrafish International Resource Center
ZMP: Zebrafish Mutation Project
ZnF: Zinc Finger
