# Supplementary material for: Zebrafish prdm12b acts independently of nkx6.1 repression to promote eng1b expression in the neural tube p1 domain
Source: Neural Dev. 2019 Feb 27;14:5. doi: 10.1186/s13064-019-0129-x (PMC6391800; doi:10.1186/s13064-019-0129-x)
Supplement: Supplementary file 1 — Sequences of oligos used to generate CRISPR guide RNAs. Detailed features of the oligos used as templates for each guide RNA. (DOCX 13 kb) [file 13064_2019_129_MOESM1_ESM.docx]

**Additional File 1. Sequences of oligos to generate single guide RNAs**

| **Target Gene** | **Target sequence ^a^** | **PAM ^b^** | **First oligo ^c^** | **Second oligo ^d^** |
| --- | --- | --- | --- | --- |
| *prdm12b* | GCTGGGGGAACACCTGTTCG | AGG | TTAATACGACTCACTATAGG GCTGGGGGAACACCTGTTCG GTTTTAGAGCTAGAAATAGCAAG | AAAAAAGCACCGACTCGGTGCCACTTTTTCAAGTTGATAACGGACTAGCCTTATTTTAACTTGCTATTTCTAGCTCTAAAAC |
| *bhlhe22* | TTCACACACAAAGATCCGGT | CGG | TTAATACGACTCACTATAGG TTCACACACAAAGATCCGGT GTTTTAGAGCTAGAAATAGCAAG | AAAAAAGCACCGACTCGGTGCCACTTTTTCAAGTTGATAACGGACTAGCCTTATTTTAACTTGCTATTTCTAGCTCTAAAAC |
| *nkx6.1* | AGTGGAGGATGCTGGTCCAG | TGG | TTAATACGACTCACTATAG AGTGGAGGATGCTGGTCCAG GTTTTAGAGCTAGAAATAGCAAG | AAAAAAGCACCGACTCGGTGCCACTTTTTCAAGTTGATAACGGACTAGCCTTATTTTAACTTGCTATTTCTAGCTCTAAAAC |

^a^ Genomic sequence targeted by the single guide RNA

^b^ Protospacer adjacent motif (PAM) sequence in the genomic DNA recognized by Cas9

^c^ Sequence of the first oligo used to assemble the guide template containing the T7 promoter sequence

^d^ Sequence of the second oligo used to assemble the guide template containing the constant region
