# Supplementary material for: Zebrafish prdm12b acts independently of nkx6.1 repression to promote eng1b expression in the neural tube p1 domain
Source: Neural Dev. 2019 Feb 27;14:5. doi: 10.1186/s13064-019-0129-x (PMC6391800; doi:10.1186/s13064-019-0129-x)
Supplement: Supplementary file 2 — Sequences of primers used to genotype mutant lines. Detailed features of primers used to genotype mutant lines. For bhlhe22, the bhlhe22–1 and bhlhe22–2 primers were used to amplify genomic DNA while the bhlhe22–3 and bhlhe22–4 primers were used to amplify cDNA. (DOCX 13 kb) [file 13064_2019_129_MOESM2_ESM.docx]

**Additional file 2. Primers used for genotyping of *prdm12b, bhlhe22 and nkx6.1* mutant fish**

| **Primer name** | **Sequence** | **Purpose** |
| --- | --- | --- |
| *prdm12b-1* | GGTTCGGCTCATCATGGGTTC | Forward primer used to genotype um318, um319 |
| *prdm12b-2* | GCAAACTGACCTCCAGAGAA | Reverse primer used with primer *prdm12b-1* |
| *prdm12b-3* | TTCCAGCTTAGTTCTGCCAAGTG | Forward primer used to genotype sa9887 |
| *prdm12b-4* | CGACCTCCAAGTTCTGTTCTT | Reverse primer used with primer *prdm12b-3* |
| *bhlhe22-1* | AGAATAAACTTGGGCGGAGAC | Forward primer used to genotype um320 |
| *bhlhe22-2* | CATTGCTTACACAGGCTGGA | Reverse primer used with primer *bhlhe22-1* |
| *bhlhe22-3* | GCATCCGACTTTCTGGAGAC | Forward primer used to genotype um320 |
| *bhlhe22-4* | GCTGGAGGTGACATTGTTGAA | Reverse primer used with primer *bhlhe22-3* |
| *nkx6.1-1* | GGTCACTGTCCTGCTTCTTG | Forward primer used to genotype um321 |
| *nkx6.1-2* | CCACACCTTGACTTGACTCTC | Reverse primer used with primer *nkx6.1-1* |
