# Supplementary material for: Zebrafish prdm12b acts independently of nkx6.1 repression to promote eng1b expression in the neural tube p1 domain
Source: Neural Dev. 2019 Feb 27;14:5. doi: 10.1186/s13064-019-0129-x (PMC6391800; doi:10.1186/s13064-019-0129-x)
Supplement: Supplementary file 3 — Sequence of mutant prdm12b, bhlhe22 and nkx6.1 alleles. The predicted amino acid sequence for each mutant allele was aligned to the corresponding wildtype sequence using Clustal Omega. (PDF 236 kb) [file 13064_2019_129_MOESM3_ESM.pdf]

A

```
prdm12b

WT          MGSVLPADALVLKAGFKQQSLALSDIITSDILHSFLYGRWRNVLGEHLFEEKTATVSPKT   60
um318       MGSVLPADALVLKAGFKQQSLALSDIITSDILHSFLYGRWRPQ---NLRSQPQNCLHRRG   57
um319       MGSVLPADALVLKAGFKQQSLALSDIITSDILHSFLYGRWRNVRGEDRHCQPQNCLHRRG   60

WT          AFTAEVLAQSFSGEVQKLSSLVLPSEVIIAQSSIPGEGLGIFSKTWIKAGTEMGPFTGRV   120
um318       PGAVILWRGPETFQLSSAK-----------------------------------------   76
um319       PGAVILWRGPETFQLSSAK-----------------------------------------   79

WT          ISPEHVDLFKNNNLMWEVFNEDGTVRYFIDASQEDHRSWMTYIKCARNEQEQNLEVVQIG   180
um318       ------------------------------------------------------------   76
um319       ------------------------------------------------------------   79

WT          SSIFYKAVETIPPDQELLVWYGNSHNTFLGIPGVPGTEEEQQKKTKTDEFHLCDTVTAAA   240
um318       ------------------------------------------------------------   76
um319       ------------------------------------------------------------   79

WT          LSTASRMRCVICHRGFNSRSNLRSHMRIHTLDKPFVCRFCNRRFSQSSTLRNHVRLHTGE   300
um318       ------------------------------------------------------------   76
um319       ------------------------------------------------------------   79

WT          RPYKCHVCQSAYSQLAGLRAHQKSARHRPANTGAVVGLQAHSPPPPQLAQVPHPASLVHH   360
um318       ------------------------------------------------------------   76
um319       ------------------------------------------------------------   79

WT          IPTMVL     366
um318       ------     76
um319       ------     79
```

B

```
bhlhe22

WT          MDRRINLGGDIFHKTLSAVSSKKMDSFRPTAGIDLSARDSQSPISCFEQNDPDPVQPGGR   60
um320       MDRRINLGGDIFHKTLSAVSSKKMDSFRPTAGIDLSARDSQSPISCFEQNDPDPVQPGGR   60

WT          AGTLGLPTGSLCVKYGESANRTSGAESSGGEQSPDDDSDERCEMMLMTDGRTTVPGAKSE   120
um320       AGTLGLPIFVCEIRRERQQDFRRGEQRRRAKPGR--------------------------   94

WT          GGKKNKEQKMLRLNINARERRRMHDLNDALDELRAVIPYAHSPSVRKLSKIATLLLAKNY   180
um320       ------------------------------------------------------------   94

WT          ILMQAQALEEMRRLVAYLNQGQAISAASLPATTALTPGLSAYEQPAGYPFPAGVAASSCP   240
um320       ------------------------------------------------------------   94

WT          DKCALFNNVTSSLCKQCTDKP     261
um320       ---------------------     94
```

C

```
nkx6.1

WT          MLAVGQMDGSRQSAFLLNTPPLAALHSMTEMKTPLYPAYPLSSTGPASSTSPTATSPNPG 60
um321       MLAVGQMDGSRQSAFLLNTPPLAALHSMTEMKTPLYPAYPLSS--------TDSYLSESR 52
um322       MLAVGQMDGSRQSAFLLNTPPLAALHSMTEMKTPLYPAYPLSSTASSILHFTDSYLSESR 60

WT          GIPVSSPGIKTSSGLSALASAQQCAIATPHGINDILSRPSVACSPAGILSGLPRFSSLSP 120
um321       WDPGLVPGDQNLQWTFSSRISPAVRDRHTSRNKRHPQPTLGGLFSRRNLVWTAPFQQPES 112
um322       WDPGLVPGDQNLQWTFSSRISPAVRDRHTSRNKRHPQPTLGGLFSRRNLVWTAPFQQPES 120

WT          PPPPGLYFSPSAAAVAVARYPKPLTELPGRTPIFWPGVMQSPHWRDARFACSPHQNSVLL 180
um321       PTASRALFQP-------------------------------------------------- 122
um322       PTASRALFQP-------------------------------------------------- 130

WT          DKDGKRKHTRPTFSGQQIFALEKTFEQTKYLAGPERARLAYSLGMTESQVKVWFQNRRTK 240
um321       ------------------------------------------------------------ 122
um322       ------------------------------------------------------------ 130

WT          WRKRHAAEMASAKKKQDSETERLKGASENEDDDDDYNKPLDPNSDDEKITQLLKKHKPNT 300
um321       ------------------------------------------------------------ 122
um322       ------------------------------------------------------------ 130

WT          ALIIHTSENESS    312
um321       ------------    122
um322       ------------    130
```
