# Supplementary figures and images for: Zebrafish prdm12b acts independently of nkx6.1 repression to promote eng1b expression in the neural tube p1 domain
Source: Neural Dev. 2019 Feb 27;14:5. doi: 10.1186/s13064-019-0129-x (PMC6391800; doi:10.1186/s13064-019-0129-x)

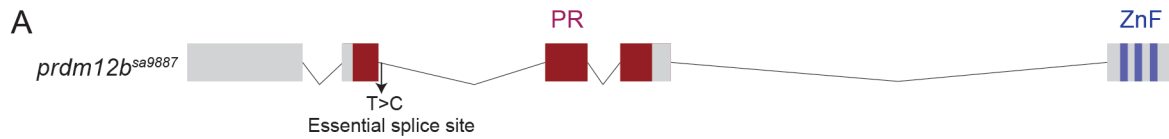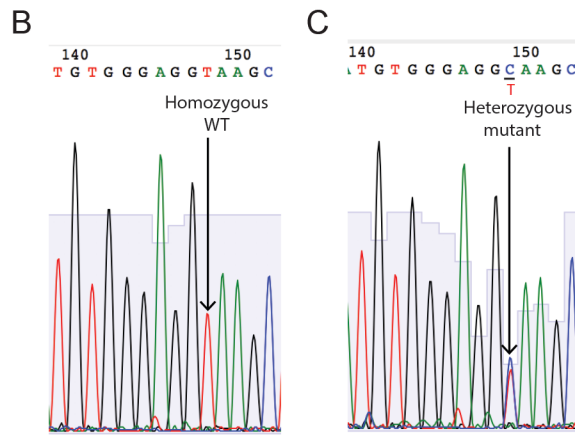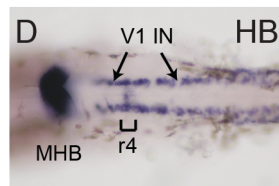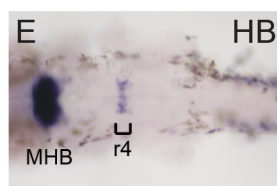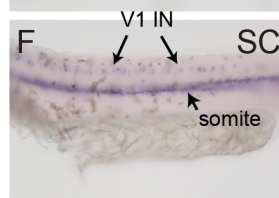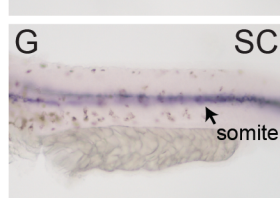

73%

27%

Supplement: Supplementary file 4 — Characterization of the prdm12bsa9887 mutant. a. Schematic showing genomic sequence of prdm12b. Exons are indicated as boxes and black lines represent introns. The PR domain and three zinc fingers (ZnF) are highlighted in dark red and blue, respectively. The black arrow indicates a single base pair change in the second intron of prdm12bsa9887. b, c. Sequence traces confirming the expected single nucleotide change in wildtype (b) versus prdm12b+/sa9887 (c) animals. d-g. eng1b expression in 24hpf embryos from a cross of prdm12b+/sa9887 animals. Embryos are shown in dorsal (d, e) or lateral (f, g) view with anterior to the left. eng1b expression is lost in 27% of embryos compared to 73% of embryos showing wildtype eng1b staining. (PDF 1962 kb) [file 13064_2019_129_MOESM4_ESM.pdf]

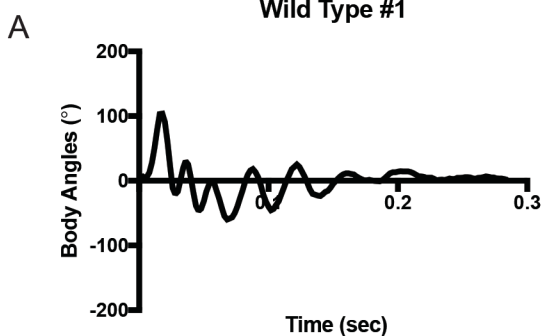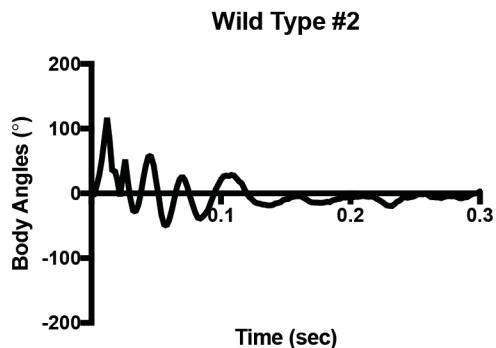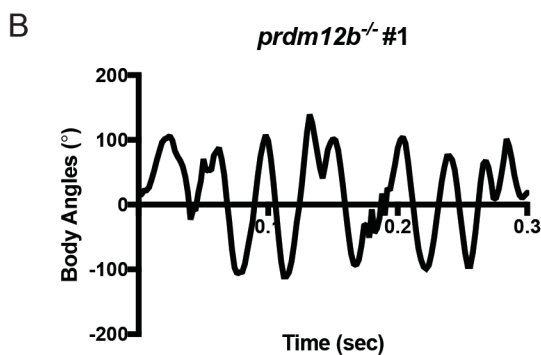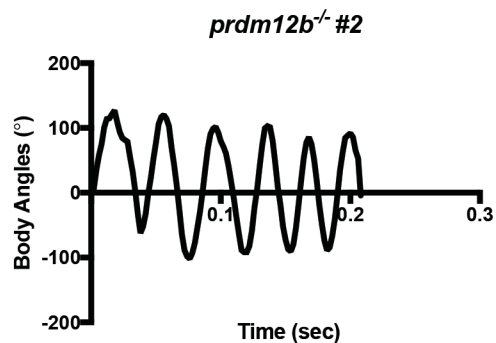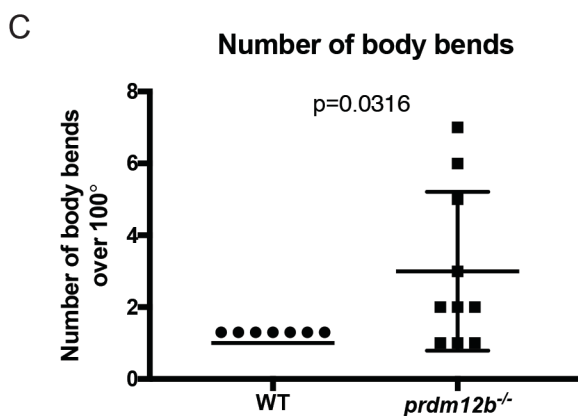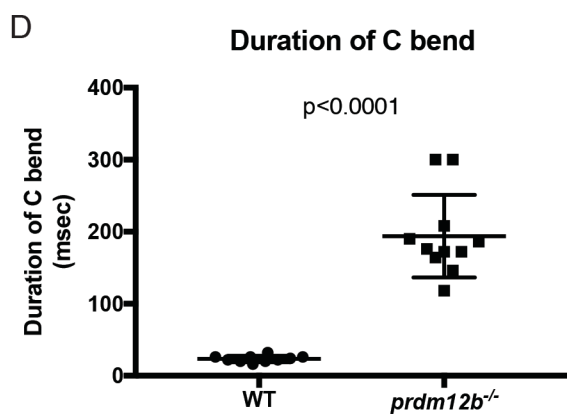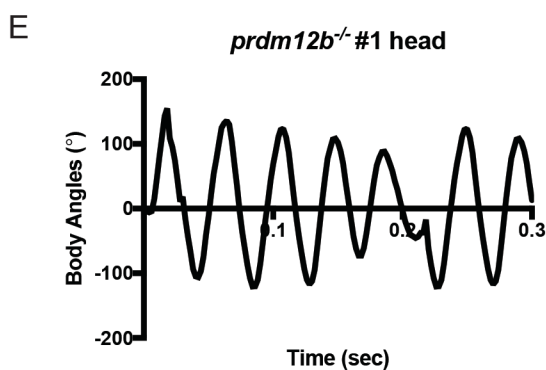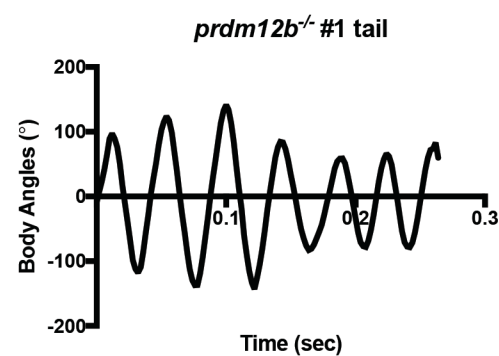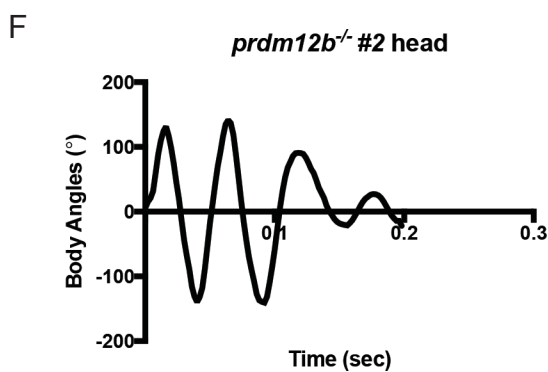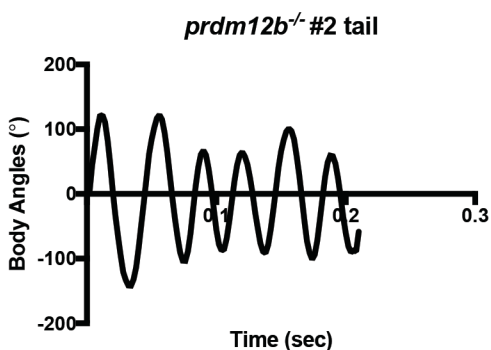

Supplement: Supplementary file 5 — Detailed analysis of the touch-evoked escape response in prdm12b mutant and wild type animals. a, b. Representative kinematic traces of individual wild type (a) and prdm12b mutant (B) animals stimulated with a head tap (from Fig. 3a, b). c. Quantification of number of body bends with an amplitude similar to the C-bend (defined as exceeding 100°; from data collected in Fig. 3a, b). d. Quantification of C bend duration (from data collected in Fig. 3a, b). e, f. Representative kinematic traces of individual prdm12b mutant animals stimulated with a head (left panels) or a tail (right panels) tap (from Fig. 3c, d). (PDF 837 kb) [file 13064_2019_129_MOESM5_ESM.pdf]

A

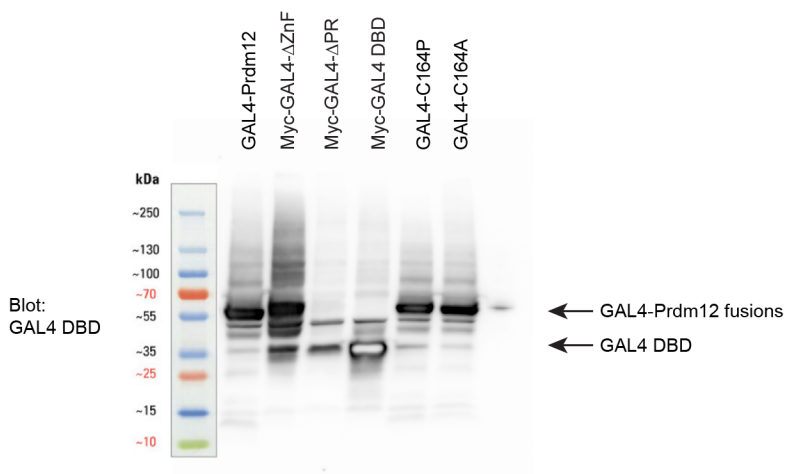

B

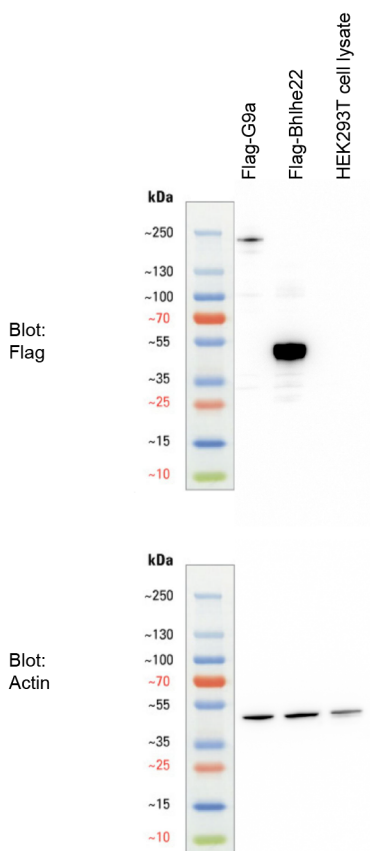

Supplement: Supplementary file 10 — Expression of GAL4DBD-Prdm12b constructs used in transfection experiments. a. Immunoblot showing expression of GAL4DBD-Prdm12b constructs in transfected HEK293T cells. All constructs are stable except Myc-GAL4-∆PR-prdm12b. b. Immunoblot showing expression of Myc-Flag-G9a and Myc-Flag-Bhlhe22 constructs in transfected HEK 293 T cells. (PDF 619 kb) [file 13064_2019_129_MOESM10_ESM.pdf]

A

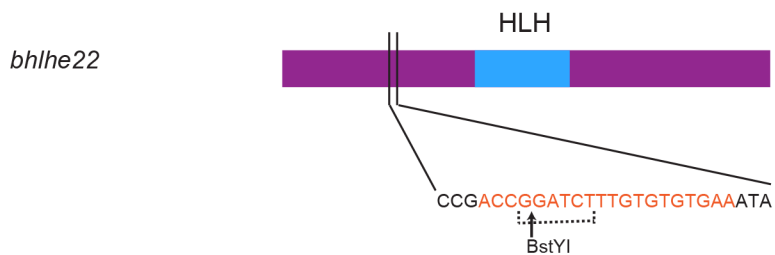

B

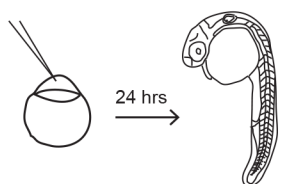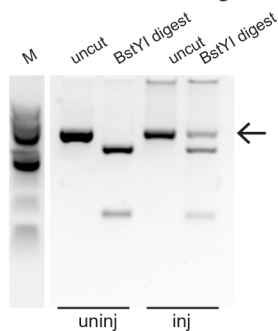

C

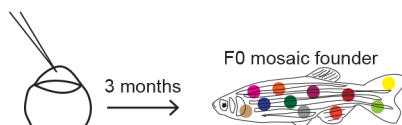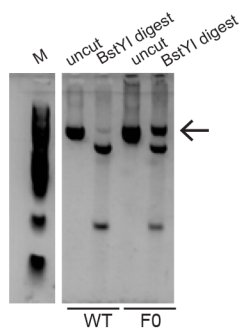

D

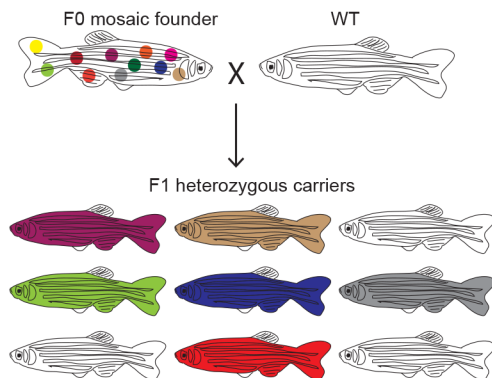

E

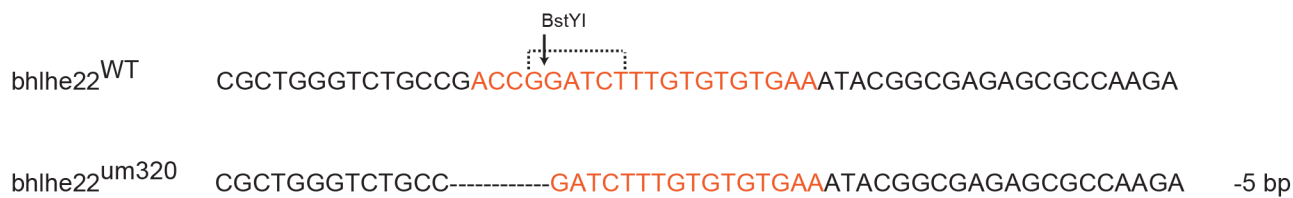

F

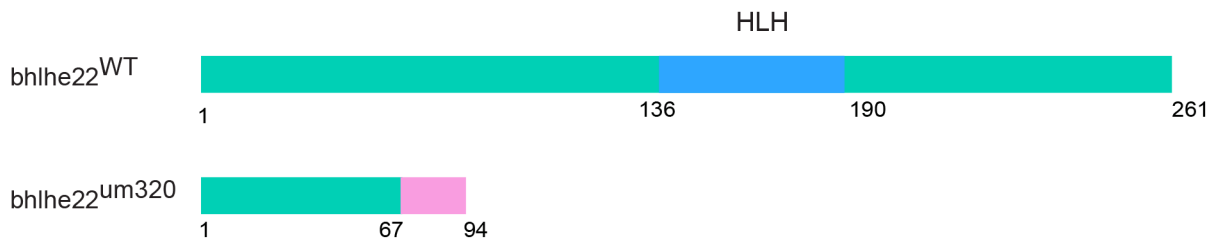

Supplement: Supplementary file 11 — Generation of bhlhe22 germline mutant. a. Schematic showing genomic sequence of bhlhe22 with the bHLH domain indicated in blue. Note that bhlhe22 is contained on a single exon. The CRISPR target sequence is shown in red with the BstYI restriction site bracketed and the black arrow indicating the BstYI cut site. b. Identification of functional guide RNAs. sgRNA and cas9 mRNA was injected into 1-cell stage embryos. Injected embryos were raised to 24hpf and BstYI digestion of PCR amplicons from pools of embryos was used to identify CRISPR-induced mutations (black arrow). c. Identification of individual F0 founders. sgRNA/cas9 injected embryos were raised to adulthood and crossed to wildtype fish. BstYI digests of PCR amplicons from pools of embryos was used to identify F0 mosaic founders (black arrow). d. Identification of F1 animals. Adult F0 mosaic founders were out-crossed to wildtype fish and the F1 offspring raised to adulthood. BstYI digests of PCR amplicons from fin clip genomic DNA was used to identify heterozygous F1 animals. e. Sequencing of F1 genomic DNA revealed the transmission of one mutant allele (um320) carrying a 5 base pair deletion (black dashes). The CRISPR target sequence is shown in red. f. Predicted amino acid sequence of mutant allele. The um320 peptide shares its first 67 amino acids with the wildtype protein before going out of frame and terminating at a premature stop codon N-terminal to the bHLH domain. (PDF 485 kb) [file 13064_2019_129_MOESM11_ESM.pdf]

A

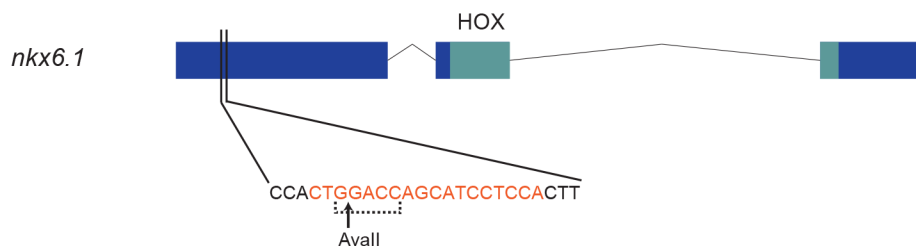

B

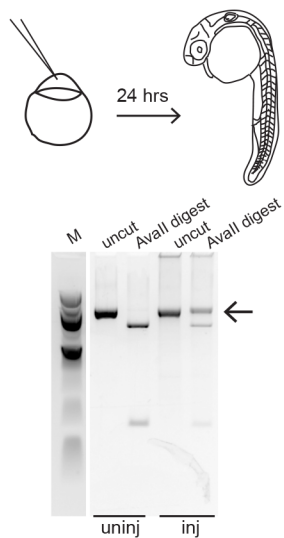

C

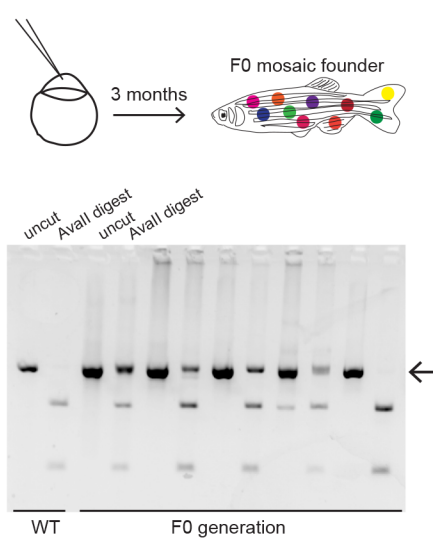

D

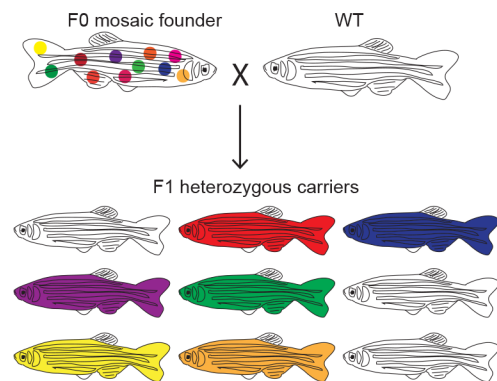

E

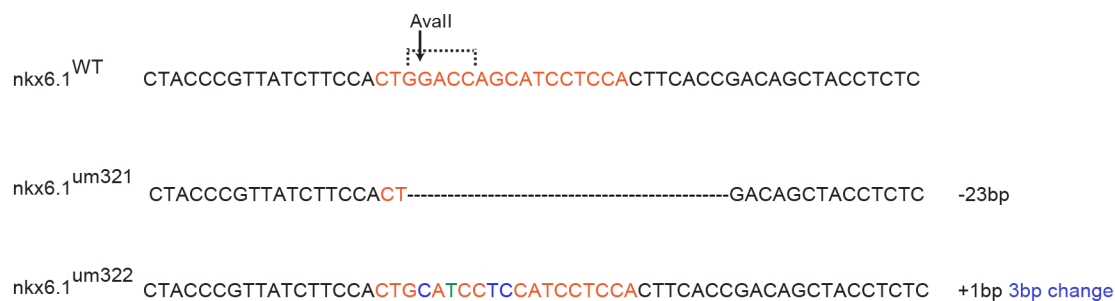

F

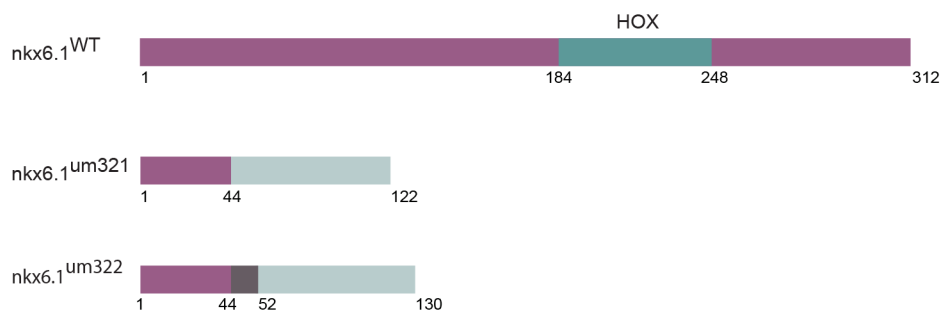

G

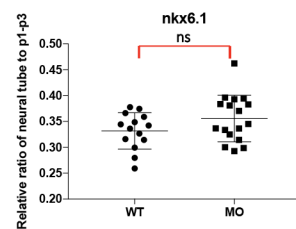

Supplement: Supplementary file 12 — Generation of germ line nkx6.1 mutants. a. Schematic showing genomic sequence of nkx6.1 with the homeodomain indicated in green. The CRISPR target sequence is shown in red with the AvaII restriction site bracketed and the black arrow indicating the AvaII cut site. b. Identification of functional guide RNAs. sgRNA and cas9 mRNA was injected into 1-cell stage embryos. Injected embryos were raised to 24hpf and AvaII digestion of PCR amplicons from pooled embryos was used to identify CRISPR-induced mutations (black arrow). c. Identification of individual F0 founders. sgRNA/cas9 injected embryos were raised to adulthood and crossed to wildtype fish. AvaII digests of PCR amplicons from pools of embryos was used to identify F0 mosaic founders (black arrow). d. Identification of F1 animals. Adult F0 mosaic founders were out-crossed to wildtype fish and the F1 offspring raised to adulthood. AvaII digests of PCR amplicons from fin clip genomic DNA was used to identify heterozygous F1 animals. e. Sequencing of F1 genomic DNA revealed the transmission of two mutant alleles (um321, um322). um321 carries a 23 base pair deletion (black dashes) while um322 carries a 1 base pair insertion (green) and 3 base pair substitutions (blue). The CRISPR target sequence is shown in red. f. Predicted amino acid sequence of mutant alleles. The um320 and um321 peptides share their first 44 amino acids with the wildtype sequence before going out of frame and terminating at a premature stop codon N-terminal to the conserved homeodomain. g. Quantification of the size (along the dorsoventral axis) of the nkx6.1 expression domain in prdm12b MO-injected embryos (data from Fig. 8). (PDF 651 kb) [file 13064_2019_129_MOESM12_ESM.pdf]
